# Deferasirox Targets TAOK1 to Induce p53-Mediated Apoptosis in Esophageal Squamous Cell Carcinoma

**DOI:** 10.3390/ijms26041524

**Published:** 2025-02-11

**Authors:** Boyang Li, Shihui Liu, Xiaowan Zhou, Dongpu Hou, Huajie Jia, Rude Tang, Yunqing Zhang, Mengqiu Song

**Affiliations:** 1 Department of Pathophysiology, School of Basic Medical Sciences, College of Medicine, Zhengzhou University, Zhengzhou 450001, China; boyangli@stu.zzu.edu.cn (B.L.);; 2 The First Clinical School of Medicine, Zhengzhou University, Zhengzhou 450001, China; 3 China-US (Henan) Hormel Cancer Institute, No. 127, Dongming Road, Jinshui District, Zhengzhou 450008, China; 4 Tianjian Laboratory of Advanced Biomedical Sciences, Institute of Advanced Biomedical Sciences, Zhengzhou University, Zhengzhou 450001, China; 5 Henan International Joint Laboratory of Cancer Chemoprevention, Zhengzhou University, Zhengzhou 450001, China; 6 State Key Laboratory of Esophageal Cancer Prevention and Treatment, Zhengzhou University, Zhengzhou 450001, China

**Keywords:** deferasirox, TAOK1, esophageal squamous cell carcinoma, apoptosis, targeted therapy

## Abstract

Esophageal squamous cell carcinoma (ESCC) is a highly aggressive malignancy with a poor prognosis and limited effective treatment options. This study investigates the therapeutic potential of Deferasirox (DFO), an iron chelator, in ESCC by targeting TAOK1, an STE20-type kinase implicated in cancer development. We demonstrate that DFO significantly inhibits the proliferation and colony formation of ESCC cells in a dose- and time-dependent manner. Mechanistic investigations reveal that DFO binds directly to TAOK1 and reduces its kinase activity. Proteomics and phosphorylated proteomic sequencing analysis further reveal that TAOK1 knocking down dramatically increased p53-mediated apoptosis. Moreover, the inhibition of TAOK1 by DFO or lenti-virus infection induces apoptosis in ESCC cells, as evidenced by the increased expression of p53, p-p53 (S15), p-p53 (S46), Puma, Noxa, and Bax, and the decreased expression of Bcl-2. Furthermore, in vivo studies using patient-derived xenograft (PDX) mouse models show that DFO treatment significantly reduces tumor volume without observable toxicity. Histological and immunohistochemical analyses confirm the down-regulation of TAOK1 and Ki-67, and the up-regulation of p53 expression in DFO-treated tumors. Our findings suggest that DFO exerts its antitumor effects in ESCC by targeting TAOK1, providing a potential therapeutic strategy for ESCC patients.

## 1. Introduction

Esophageal cancer (EC) is a highly aggressive malignancy originating from the esophageal epithelium, characterized by rapid progression and a low survival rate [1,2]. Esophageal squamous cell carcinoma (ESCC), which accounts for up to 90% of all esophageal cancer cases, is the predominant histological type worldwide [3]. Due to its aggressive nature and the lack of obvious early symptoms, early detection remains challenging, and most patients are diagnosed at advanced stages, where curative treatment options are limited [4]. Despite advancements in therapeutic strategies, including surgery, radiotherapy, and chemotherapy, the prognosis for ESCC patients remains poor, with a low five-year survival rate [3]. Currently, there are no approved targeted therapies for ESCC. Therefore, identifying new molecular targets and developing small molecule inhibitors that can significantly suppress ESCC growth are of critical importance for the treatment of this disease.

Thousand-and-one amino acid protein kinases (TAOKs) include TAOK1, TAOK2, and TAOK3, which act as upstream regulators of the mitogen-activated protein kinase (MAPK) cascade [5,6]. Recent studies have shown a growing research focus on TAOK1 in cancer malignancies. Several studies have demonstrated that TAOK1 possesses oncogenic properties in non-small cell lung cancer (NSCLC), hepatocellular carcinoma (HCC), and ESCC [7,8,9]. It is reported that the inhibition of TAOKs using newly synthesized compounds 43 and 63 delays cell mitosis in centrosome-amplified breast cancer cells [10]. Moreover, targeting TAOK1 by resveratrol dramatically inhibits ESCC growth in vitro and in vivo [9]. Additionally, corylifol A ameliorates muscle atrophy by inhibiting the TAOK1/p38-MAPK/FoxO3 pathway in cancer cachexia [11]. However, functional studies on targeting TAOK1 in ESCC are in their infancy, and its pathophysiological mechanism requires further exploration.

Deferasirox (DFO), an iron chelator, which has been approved by the FDA for the treatment of iron overload, has recently been shown to possess both anti-cancer and antimicrobial properties [12,13,14,15]. DFO significantly inhibited cervical cancer by suppressing Akt and the MEK/ERK signaling pathway as well as decreased the levels of ferritin in serum and tumor tissues at a relatively high concentration of more than 50 μM [16]. Moreover, a DFO-loaded specific M1 macrophage-derived exosome (M1Exo)-based drug delivery system increased the chemosensitivity of gemcitabine (GEM) in pancreatic cancer via depleting iron [16]. In ulcerative colitis (UC), the utilization of DFO relieved inflammation by inhibiting ferroptosis and improving intestinal microbiota [17]. In esophageal cancer, DFO was shown to effectively deplete iron from esophageal tumor cells to reach a growth suppression effect [18]. However, the molecular mechanism of DFO in ESCC, beyond its iron-chelating function, has not yet been elucidated.

This study aims to thoroughly investigate the antitumor effects of DFO in ESCC cells and patient-derived xenograft (PDX) tumors both in vitro and in vivo, focusing on mechanisms independent of iron chelation. Furthermore, we reveal the potential of DFO as a targeted therapeutic agent in ESCC by examining its interaction with TAOK1 and associated downstream signaling pathways. These findings offer new insights into the functional mechanisms of DFO in ESCC and suggest TAOK1 as a promising target for novel therapeutic strategies.

## 2. Results

### 2.1. Deferasirox Significantly Inhibits ESCC Cell Proliferation and Colony Formation

**Figure 1:**
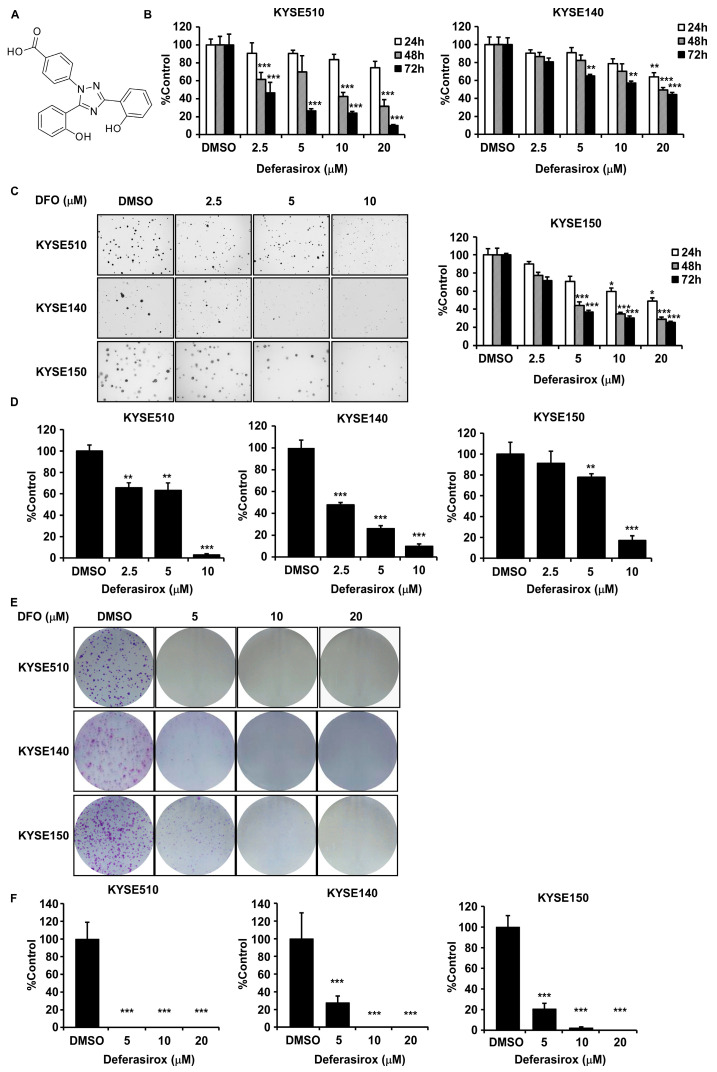
Deferasirox significantly inhibits ESCC cell proliferation and colony formation. (**A**) Chemical structure of DFO. (**B**) Inhibitory effects of DFO on KYSE510, KYSE140, and KYSE150 ESCC cells were measured using MTT assays at 24, 48, and 72 h. Vehicle, 2.5, 5, 10, and 20 μM of DFO were adopted for measurements. Effects of DFO on anchorage-independent ESCC cells growth were evaluated. Vehicle, 2.5, 5, and 10 μM of DFO were used for the assay. Representative photographs illustrating the effects of DFO on anchorage-independent cell growth (**C**) and analysis data (**D**) are shown. Effects of DFO on the foci formation abilities of ESCC cells were evaluated. Vehicle, 5, 10, and 20 μM DFO were used for these experiments. Representative photographs illustrating the effects of DFO treatment on foci formation (**E**) and analysis data (**F**) are shown. Data are shown as means ± SD. Asterisks (* *p* < 0.05, ** *p* < 0.01, and *** *p* < 0.001) indicate a significant decrease in cell growth or colony number in DFO-treated compared to the vehicle-treated group.

Our previous study identified the oncogenic potential of TAOK1 kinase and its potential specific inhibitors [9]. In this project, we continued to search for the inhibitor of TAOK1 from the TargetMol L1010-FDA Approved & Pharmacopeial Drug Library, which contains 3158 compounds, using a virtual screening docking method (Supplementary Figure S1A). Meanwhile, the off-target effects on TAOK2 and TAOK3 of the potential candidates were evaluated based on their docking scores (Supplementary Figure S1B and Supplementary file-docking list). The compound deferasirox (Structure shown in Figure 1A) was ultimately confirmed as the most promising specific inhibitor of TAOK1. Interestingly, DFO has predominantly been studied in the context of ferroptosis in most anti-cancer mechanism research, with its effective concentration generally being quite high (~100 µM). However, in our study, we limited the maximum concentration of DFO to 20 µM in order to investigate its anti-cancer effects as a targeted therapeutic agent. DFO (0–20 μM) significantly inhibited KYSE510, KYSE140, and KYSE150 ESCC cells proliferation in a dose- and time-dependent way, with minimal cytotoxicity on normal esophageal epithelial cells (SHEE) (Figure 1B and Supplementary Figure S2A). Moreover, foci formation and anchorage-independent cell growth assays showed that DFO significantly suppressed ESCC colony formation (Figure 1C–F). These results indicated that DFO significantly inhibited ESCC cell proliferation and colony formation in vitro at low concentrations.

### 2.2. Deferasirox Directly Targets TAOK1 to Inhibit Its Kinase Activity

**Figure 2:**
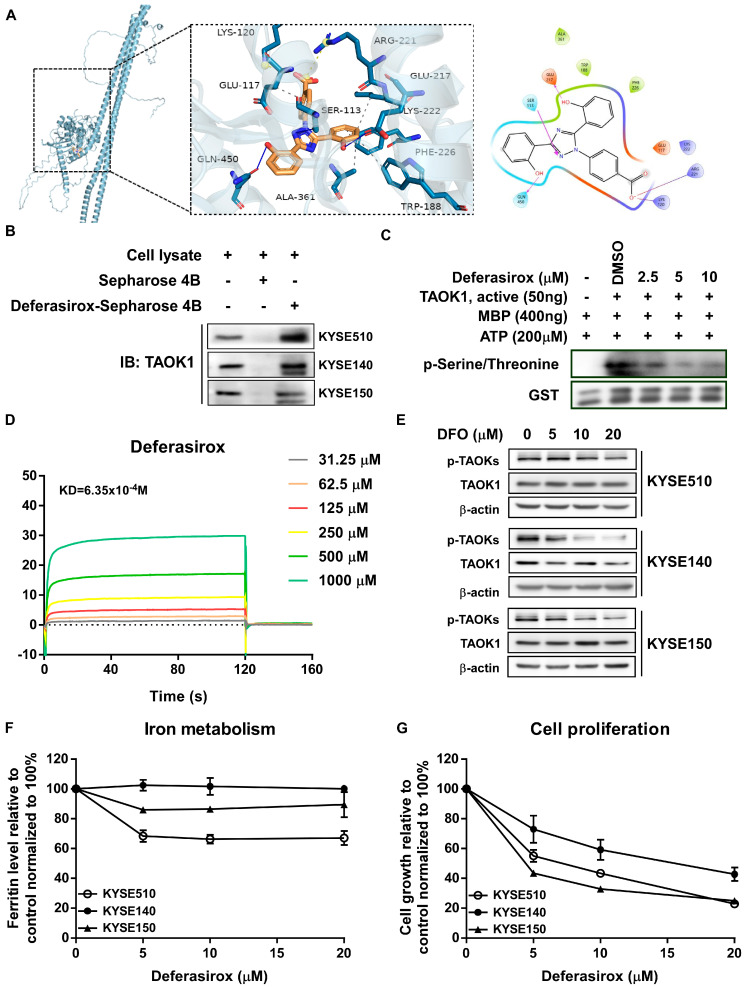
**Deferasirox directly targets TAOK1 to inhibit its kinase activity.** (**A**) Computational docking model of DFO binds with TAOK1. (**B**) The binding of DFO to TAOK1 in KYSE510, KYSE140, and KYSE150 cell lysates were determined using DMSO-/DFO-conjugated sepharose 4B beads. (**C**) The effect of DFO on TAOK1 kinase activity was measured by an in vitro kinase assay. MBP-GST was used as the substrate of TAOK1 kinase. (**D**) The DFO binding–dissociation equilibrium (KD) to TAOK1 was measured by SPR assays. (**E**) The expression of p-TAOKs and TAOK1 post DFO (vehicle, 5, 10, and 20 μM) applications were detected using Western blot. (**F**) Ferritin levels which illustrate iron metabolism in ESCC cells after DFO treatment were measured by the ELISA method. (**G**) The percentage of cell growth after DFO treatment was determined by cell proliferation assay. Data are shown as means ± SD.

To further confirm the target of DFO to TAOK1, the structure of DFO was docked with TAOK1. Computational docking analysis predicted that DFO could potentially interact with TAOK1 through its ATP-binding pocket (Figure 2A). To validate these findings, we performed Sepharose 4B pull-down assays in ESCC cells. The results indicated that DFO binds to TAOK1 in ESCC cells (Figure 2B). Furthermore, in vitro kinase assays showed that DFO treatment significantly reduced TAOK1 kinase activity (Figure 2C). Additionally, SPR assays demonstrated a strong binding–dissociation equilibrium between DFO and TAOK1, with a KD value calculated to be 6.35 × 10^−4^ M (Figure 2D). Moreover, DFO treatment significantly decreased TAOK1 kinase activity by suppressing p-TAOK expression in ESCC cells (Figure 2E). Iron metabolism assays and cell proliferation assays further confirmed that the inhibitory effects of DFO on ESCC and SHEE cells were not related to iron chelation but were instead attributed to its potential targeted therapeutic effect (Figure 2F,G and Supplementary Figure S2C,D). In summary, our data suggest that DFO significantly inhibits ESCC growth by targeting TAOK1 kinase activity, rather than through iron chelation.

### 2.3. Targeting TAOK1 Effectively Attenuates ESCC Growth

**Figure 3:**
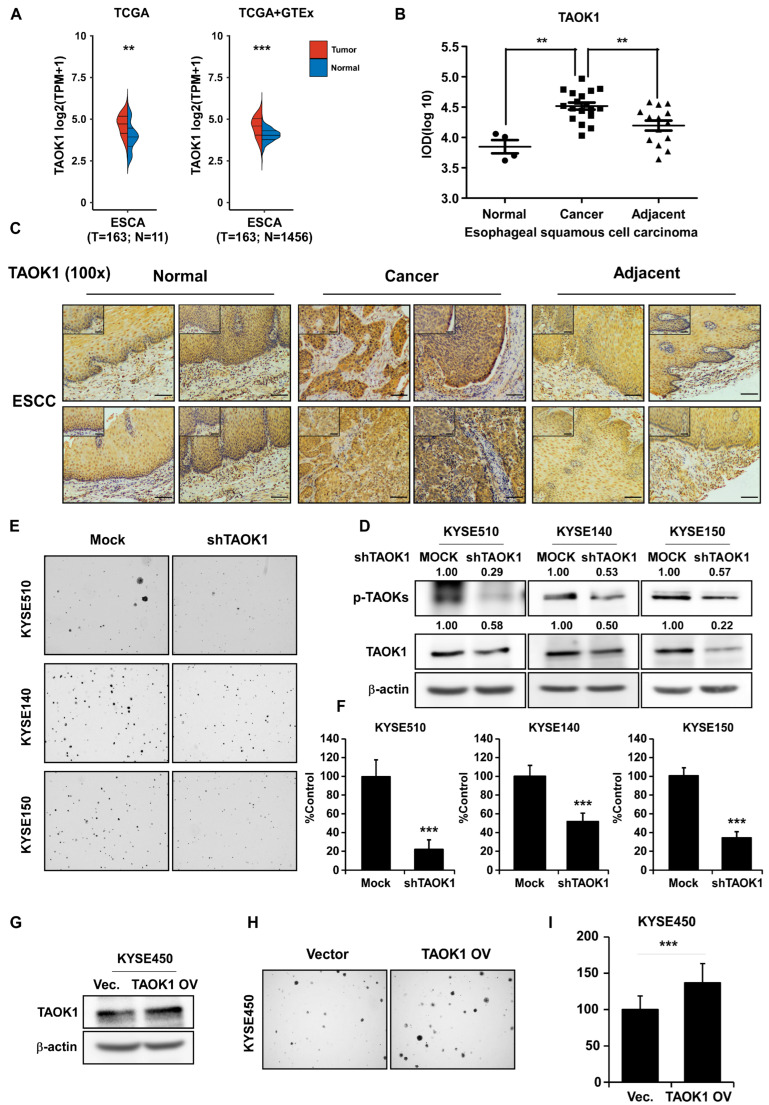
**TAOK1 acts as an onco-protein in promoting ESCC growth.** (**A**) The *TAOK1* mRNA levels in normal esophageal tissues and ESCC samples from the TCGA and TCGA + GTEx databases were retrieved and analyzed. (**B**) TAOK1 expression in patients with ESCC samples was measured by IHC analysis using a tissue microarray. Asterisks (** *p* < 0.01) indicate a significant increase in TAOK1 expression in ESCC cancer tissues compared to normal or adjacent tissues. (**C**) Representative photographs illustrating IHC staining of the ESCC tissue microarray are shown (100× magnification). (**D**) Expression of TAOK1 and p-TAOKs in KYSE510, KYSE140, and KYSE150 cell lines expressing Mock or shTAOK1 was determined by Western blot. (**E**,**F**) Anchorage-independent cell growth was assessed in KYSE510, KYSE140, and KYSE150 cell lines expressing Mock or shTAOK1, and the results were statistically analyzed. (**G**) Expression of TAOK1 in KYSE450 cells expressing Vector or TAOK1 was measured using Western blot. (**H**,**I**) Anchorage-independent cell growth was assessed in KYSE450 cells expressing Vector or TAOK1, and the results were statistically analyzed. β-actin was used as an internal reference in Western blot. Data are shown as means ± SD. Asterisks (** *p* < 0.01, and *** *p* < 0.001) indicate a significant change in TAOK1 expression or cell growth.

To validate the inhibitory effect of DFO through targeting TAOK1, TAOK1 transcript expression data from normal esophageal tissues and ESCC samples were retrieved from TCGA and GTEx databases. TAOK1 transcript expression was significantly elevated in ESCC samples (n = 163) compared to normal tissues (n = 11 or 1456) (Figure 3A). IHC experiments using a tissue array from ESCC patients were then performed to quantify TAOK1 protein expression in normal, adjacent, and cancerous tissue samples. The data indicated that TAOK1 was highly expressed in ESCC tissues compared with normal and adjacent tissues (Figure 3B,C), emphasizing the importance of targeting TAOK1 in ESCC. Furthermore, a TAOK1 knockdown assay in KYSE510, KYSE140, and KYSE150 cells using lenti-virus infection was conducted. Knockdown of TAOK1 significantly suppressed its protein expression and phosphorylation, and inhibited cell proliferation (Figure 3D–F). However, overexpression of TAOK1 in KYSE450 cells dramatically enhanced cell proliferation (Figure 3G–I). These data demonstrate that targeting TAOK1 can attenuate the malignant growth of ESCC cells.

### 2.4. TAOK1 Knockdown Elevates p53-Mediated Apoptosis in ESCC

**Figure 4:**
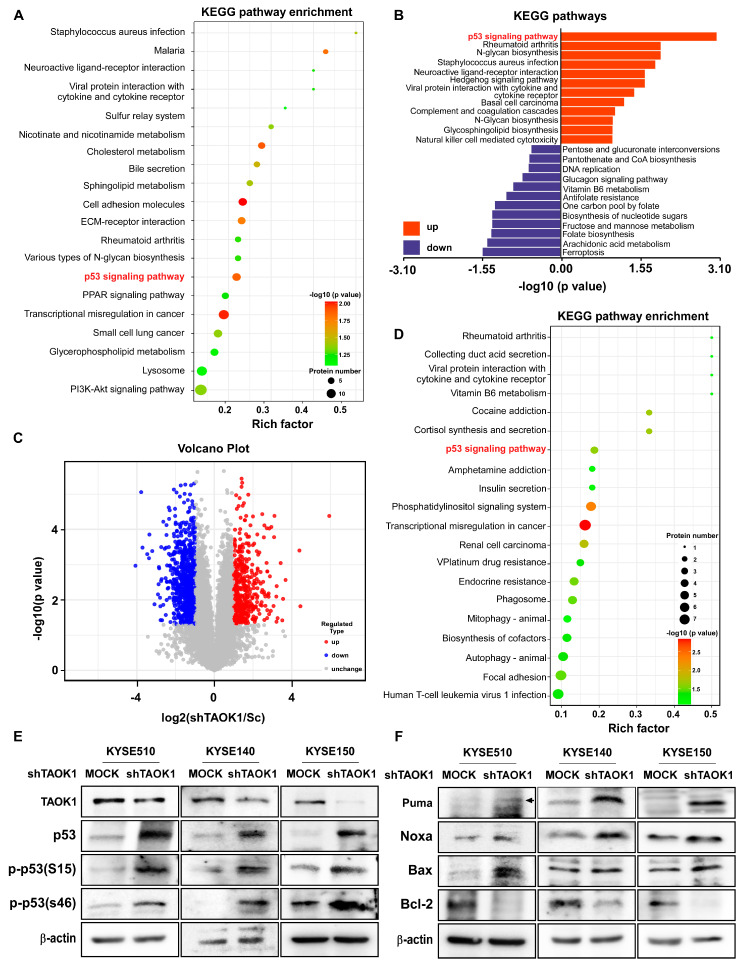
**p53-mediated apoptosis is dramatically activated following the knockdown of TAOK1.** (**A**) Proteomics sequencing and KEGG enrichment analysis of ESCC cells expressing Mock and shTAOK1 were performed. (**B**) The up-regulated and down-regulated signaling pathways following the knockdown of TAOK1 are shown. (**C**) A volcano plot indicating the up-regulated and down-regulated phosphorylated genes following the knockdown of TAOK1 are shown. (**D**) Phosphorylated proteomics sequencing and KEGG enrichment analysis of ESCC cells expressing Mock and shTAOK1 were performed. (**E**) Expression of TAOK1, p53, p-p53(S15), and p-p53(s46) in KYSE510, KYSE140, and KYSE150 cell lines expressing Mock or shTAOK1 was measured by Western blot. (**F**) Expression of Puma, Noxa, Bax, and Bcl-2 in KYSE510, KYSE140, and KYSE150 cell lines expressing Mock or shTAOK1 was measured by Western blot. β-actin was used as an internal reference.

To investigate the impact of targeting TAOK1 on the subsequent changes in cellular behavior, we conducted multi-omics analyses to examine the changes in gene expression and signaling pathway action following TAOK1 knocking down. In proteomic sequencing data, KEGG pathway enrichment analysis revealed a significant alteration in the p53 signaling pathway upon TAOK1 knockdown, suggesting a close association between TAOK1 and the p53-mediated apoptosis pathway (Figure 4A,B, and Supplementary Figure S3). Furthermore, the volcano plot demonstrated significant changes in phosphorylated protein expression levels, reflecting the cellular functional protein expression alterations induced by TAOK1 knockdown (Figure 4C). Phosphoproteomic analysis indicated that TAOK1 knockdown led to changes in cellular phosphorylation levels, affecting proteins associated with the p53 signaling pathway (Figure 4D). In TAOK1-knockdown cells, there was an increase in the expression of p53 and its phosphorylated forms, p-p53(S15) and p-p53(S46). Additionally, the expression of pro-apoptotic proteins, including Bax, Puma, and Noxa, was up-regulated, whereas the anti-apoptotic protein Bcl-2 was down-regulated (Figure 4E,F). These results collectively suggest that TAOK1 modulates the expression and phosphorylation level of p53, thereby promoting p53-mediated apoptosis in ESCC cells.

### 2.5. DFO Treatment Promotes Apoptosis in ESCC Cells by Inhibiting TAOK1

**Figure 5:**
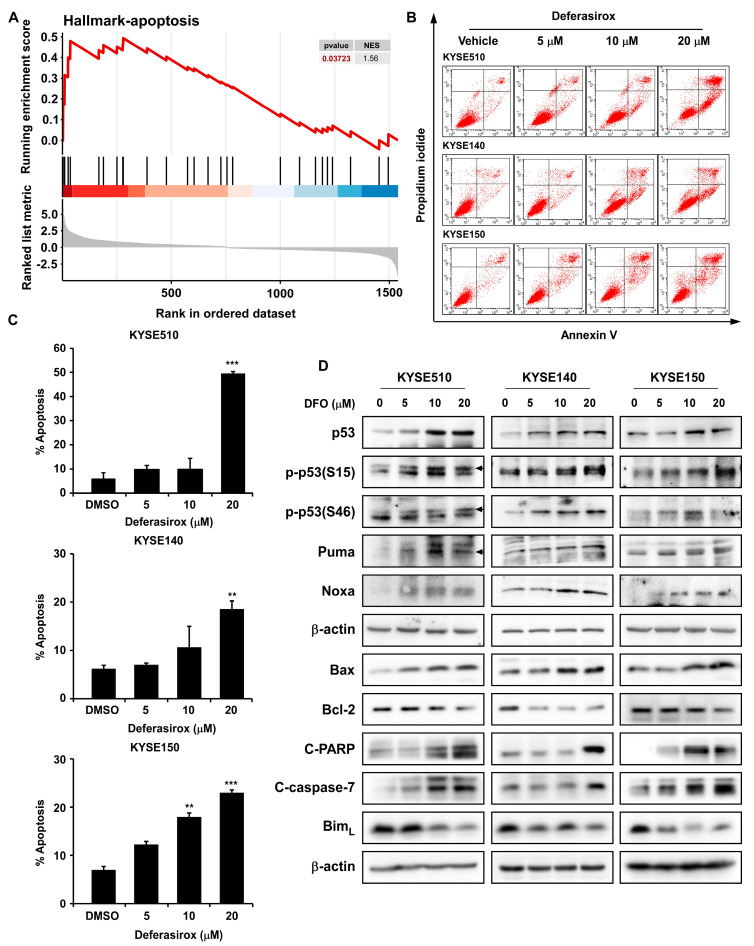
**Deferasirox treatment significantly elevated p53-mediated apoptosis in ESCC cells.** (**A**) The apoptosis signaling pathway was up-regulated following TAOK1 inhibition via GSEA analysis of the proteomics data. (**B**,**C**) Cell apoptosis was evaluated by flow cytometry following DFO treatment. The percentage of apoptosis was graphed and statistically analyzed. (**D**) Expression of p53, p-p53(S15), p-p53(s46), Puma, Noxa, Bax, cleaved PARP, cleaved caspase-7, Bcl-2, and Bim_L_ in KYSE510, KYSE140, and KYSE150 cell lines after DFO application were measured by Western blot. β-actin was used as an internal reference. Data are shown as means ± SD. Asterisks (** *p* < 0.01, and *** *p* < 0.001) indicate a significant change in DFO-induced cell apoptosis.

To investigate the relationship between DFO and cell apoptosis, we performed proteomic analysis, Western blot, and flow cytometry. Proteomic analysis of gene expression changes following TAOK1 knockdown revealed significant enrichment of the Hallmark-apoptosis pathway, which indicates the up-regulation of cell apoptosis (Figure 5A). Annexin V/PI staining followed by flow cytometry analysis was conducted on KYSE510, KYSE140, and KYSE150 cells to validate the apoptotic effects of DFO treatment. As a result, DFO treatment significantly increased the proportion of apoptotic cells in a dose-dependent manner (Figure 5B,C). However, no apoptosis was observed in SHEE cells after the application of DFO, further indicating the low cytotoxicity of DFO (Supplementary Figure S2B). Additionally, DFO treatment up-regulated the expressions of p53, p-p53(S15), and p-p53(S46). Moreover, the expression of pro-apoptotic proteins (Puma, Noxa, Bax, cleaved PARP, and cleaved caspase-7) was elevated as well, while the expression of the anti-apoptotic protein Bcl-2 was dramatically inhibited after DFO treatment (Figure 5D). These results indicate that DFO induces apoptosis in ESCC cells by suppressing TAOK1 and activating the p53-mediated signaling pathway.

### 2.6. Deferasirox Inhibits ESCC PDX Growth In Vivo

**Figure 6:**
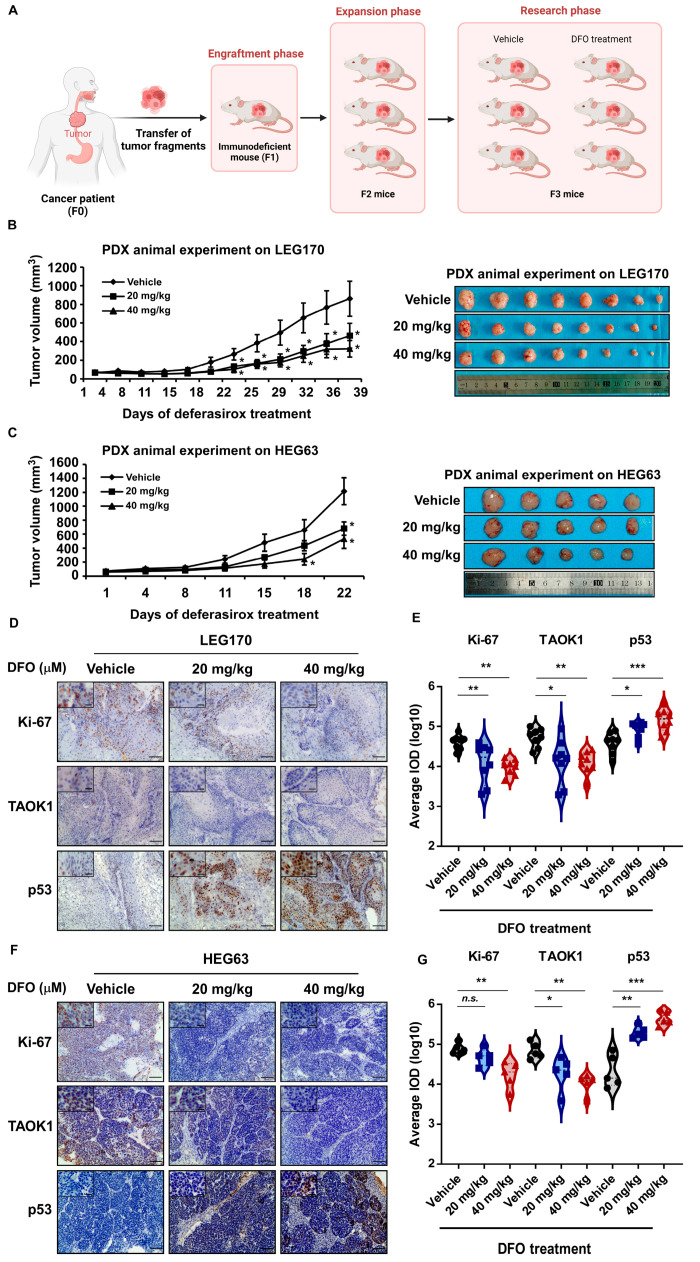
**Deferasirox inhibited ESCC PDX growth in vivo.** (**A**) Diagram of ESCC PDX generation and expansion (created by Biorender). (**B**) Tumor growth curves and images illustrating the inhibitory effects of vehicle, 20 mg/kg, and 40 mg/kg DFO on the LEG170 ESCC PDX model. (**C**) Tumor growth curves and images illustrating the inhibitory effects of vehicle, 20 mg/kg, and 40 mg/kg DFO on the HEG63 ESCC PDX model. (**D**,**E**) Representative photographs and statistical analysis results illustrating IHC staining of Ki-67, TAOK1, and p53 on LEG170 ESCC PDX tumor slides are shown (100× magnification). (**F**,**G**) Representative photographs and statistical analysis results illustrating IHC staining of Ki-67, TAOK1, and p53 on HEG63 ESCC PDX tumor slides are shown (100× magnification). Data are shown as means ± SE/SD. Asterisks (* *p* < 0.05, ** *p* < 0.01, and *** *p* < 0.001) indicate a significant change in tumor growth or indicate protein expression. n.s. indicates no significant change in the comparison.

To explore the effect of DFO on tumor growth in vivo by targeting TAOK1, two ESCC patient-derived xenograft (PDX) mouse models were carried out (Figure 6A). DFO significantly inhibited tumor growth in ESCC PDX models by suppressing TAOK1 and activating p53. In the LEG170 and HEG63 PDX models, tumor-bearing mice were daily treated with vehicle, 20 mg/kg, or 40 mg/kg DFO. Tumor volumes were significantly reduced in the DFO-treated groups in a dose-dependent manner compared to the vehicle-treated group (Figure 6B,C). Representative images of excised tumors further confirmed the suppressive effect of DFO on tumor growth (Figure 6B,C). IHC and quantitative analysis revealed that DFO treatment significantly decreased the expression of Ki-67, a proliferation marker, and TAOK1 in tumor tissues from both the LEG170 and HEG63 models. In contrast, the expression of p53, a key regulator of apoptosis, was significantly increased following DFO treatment (Figure 6D–G). Moreover, the application of DFO in mice exhibited minimal or no side effects, as indicated by the body weight, liver weight, spleen weight, and pathological changes observed in the H&E staining of the liver and spleen (Supplementary Figure S4). To determine whether the antitumor function of DFO in vivo is independent of its iron-chelating activity, ELISA experiments were conducted to measure ferritin and transferrin levels in mouse serum after DFO treatment. The data showed that both ferritin and transferrin concentrations did not significantly change following DFO application in the two PDX animal models, further supporting the targeting of TAOK1 by DFO in ESCC (Supplementary Figure S5). These results indicate that DFO suppresses tumor progression in ESCC PDX models by targeting TAOK1 and enhancing p53 activity, providing a potential therapeutic strategy for treating ESCC.

## 3. Discussion

**Figure 7:**
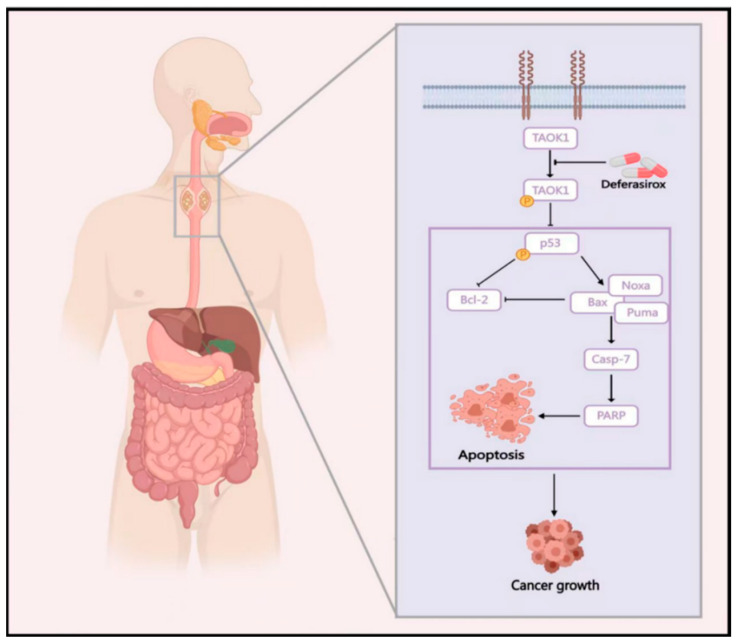
**Graphic diagram of DFO targeting TAOK1 to inhibit ESCC growth.** DFO directly targets TAOK1 to suppress its phosphorylation and activation, thereby inducing p53-mediated cell apoptosis in ESCC. Consequently, DFO significantly attenuated ESCC growth both in vitro and in vivo. This diagram was created with MedPeer (medpeer.cn).

ESCC is a highly aggressive malignancy with limited treatment options and poor survival outcomes. Despite progress in surgery, chemotherapy, and radiotherapy, the lack of effectively targeted therapies remains a critical issue in clinical practice [19]. In this study, we identified TAOK1 as a potential therapeutic target in ESCC, and Deferasirox, an FDA-approved iron chelator, exerted significant antitumor effects by inhibiting TAOK1 independent of its iron-chelating function. Moreover, DFO significantly inhibited ESCC growth and promoted p53-mediated apoptosis in vitro and in vivo by directly targeting TAOK1 (Figure 7). These findings provide new insights into the molecular mechanisms driving ESCC progression and offer a potential therapeutic strategy for clinical application.

Our previous research has demonstrated that TAOK1 was overexpressed in ESCC patient samples and acted as an onco-protein to promote ESCC malignancy [9]. In this study, we further confirmed the expression and molecular function of TAOK1 in ESCC, and the findings are consistent with our previous report. Moreover, a structure-based virtual screening approach for TAOK1 was established and validated [20]. Accordingly, a computational docking analysis was performed to screen the FDA-approved drug bank for potential TAOK1 inhibitors. To minimize off-target effects on TAOK2 and TAOK3, an MM-GBSA rescoring was conducted, allowing us to select compounds that exhibit high affinity for TAOK1 and low affinities for TAOK2 and TAOK3. Ultimately, we demonstrated that DFO, identified as the most promising inhibitor of TAOK1, directly inhibited its kinase activity by binding to the ATP-binding pocket. This mechanism closely resembles the mode of action described for ATP-competitive kinase inhibitors in previous studies [21].

Interestingly, DFO is an established iron chelator, which has proven safety and effectiveness in patients with transfusion-dependent iron overload [22]. Moreover, DFO exhibits a suppressive effect on ferroptosis in diseases such as myocardial ischemia–reperfusion injury [23]. In addition, iron chelators are considered as anti-cancer agents [24]. Specifically, DFO has been reported to suppress cervical cancer both in vitro and in vivo by reducing iron deposits and reactive oxygen species (ROS) levels [16]. In addition, DFO has demonstrated anti-cancer activity or has potentiated the effects of chemotherapy in gastric and pancreatic cancers, either dependent or independent of its iron-chelating function [25,26]. Therefore, DFO may hold strong potential for suppressing cancer malignancy beyond its role in iron metabolism. In this study, we showed that DFO dramatically inhibited ESCC cells and PDX tumor growth by directly binding to and inhibiting TAOK1 kinase activity, rather than through its iron-chelating properties. Mechanistically, DFO targeted TAOK1 to induce p53-mediated apoptosis, thereby suppressing ESCC. These findings are consistent with earlier studies that link TAOK1 to apoptosis resistance [27].

Recent reports indicate that iron chelation therapy induces immunogenic stress responses in ovarian cancer cells, thereby delaying metastatic disease progression and enhancing the effects of first-line chemotherapy [28]. In addition to their use as single agents, iron chelators combined with other anti-cancer therapies have also demonstrated synergistic effects [29]. Similarly, combining DFO with other existing therapies, such as chemotherapy or radiotherapy, offers promising avenues for enhancing therapeutic outcomes. It has been shown that DFO can improve the efficacy of radiation therapy [30]. Moreover, a dual delivery system of DFO and doxorubicin hydrochloride (DOX) can enhance antigen presentation and T-cell infiltration, ultimately contributing to tumor remission [31]. Consequently, further research on the anti-cancer potential of DFO should focus on optimizing combination regimens and strategies to maximize clinical benefits.

## 4. Materials and Methods

### 4.1. Reagents and Cells

Deferasirox (CAS No.: 201530-41-8) was purchased from MedChemExpress Co., LTD. (Monmouth Junction, NJ, USA). Active TAOK1 kinase (T0453) and GST-tagged MBP protein (SRP5205) were purchased from MRECK (Kenilworth, NJ, USA). Primary antibodies for the following protein markers were purchased from Cell Signaling Technology (Danvers, MA, USA): p53 (#2527), p-p53 (Ser15) (#9284), p-p53 (Ser46) (#2521), cleaved PARP (#5625), cleaved caspase-7 (#9491), Bax (#5023), and Bim (#2933). Antibodies to detect p-TAOKs (ab124841) and Bcl-2 (ab182858) were obtained from Abcam (Cambridge, Cambridgeshire, UK). Anti-TAOK1 (26250-1-AP), Anti-Puma (55120-1-AP), and Anti-Noxa (17418-1-AP) were obtained from Proteintech (Chicago, IL, USA). Mouse anti-β-actin antibody (#AF7018), goat anti-rabbit antibody (#S0001), and goat anti-mouse antibody (#S0002) were purchased from Affinity Biosciences Company (Cincinnati, OH, USA).

The human esophageal cancer cell lines KYSE510, KYSE140, KYSE150, and KYSE450 and Lenti-293T cells were purchased from the Type Culture Collection of the Chinese Academy of Sciences (Shanghai, China) and maintained in a humidified incubator at 37 °C under a 5% CO_2_ atmosphere. The SHEE-immortalized human normal esophageal epithelial cell line was donated by Dr. Enmin Li of the Shantou University Medical College. Cell lines used in this study were cultured in RPMI-1640 or Dulbecco’s modified Eagle’s medium (DMEM) supplemented with penicillin (100 units/mL), streptomycin (100 μg/mL), and 10% FBS (BI, M.P. Western Galilee, Israel).

### 4.2. Cell Proliferation Measurement

For MTT assay, cells (1.5 × 10^3^ cells per well) were seeded in 96-well plates and incubated for 24 h. Vehicle or the indicated concentration gradient of DFO, diluted in complete growth media, was then applied to cells. After the indicated time points, the MTT medium was aspirated and replaced in 100 μL DMSO (≥99.7%, MilliporeSigma, Burlington, MA, USA). Formazan crystals were then dissolved by gentle shaking, and the absorbance was detected at 570 nm and 620 nm. For foci formation assay, cells (1.5 × 10^3^ per well) were suspended in complete RPMI-1640 medium and seeded in 6-well plates. Vehicle or the indicated concentration gradient of DFO were applied to the cells after incubation for 24 h. Cells were maintained at 37 °C in a 5% CO_2_ incubator for 1 week. The cultures were changed to fresh media containing different concentrations of DFO every other day. Finally, the plates were washed with PBS and the foci were fixed and visualized with 0.5% crystal violet solution. Pictures were taken by MINISpace2000 (Tanon, Shanghai, China) and foci numbers were quantified by the Image-Pro Plus software (v.6.1) program (Media Cybernetics, Rockville, MD, USA).

### 4.3. Anchorage-Independent Cell Growth Assay

Cells (8 × 10^3^ per well) suspended in complete medium were suspended in 0.3% agar mix supplemented with vehicle, 5, 10, or 20 μM DFO in a top layer over a base layer of 0.5% agar supplemented with the same concentration of DFO as the top layer. The cultures were maintained at 37 °C in a 5% CO_2_ incubator for 3 weeks. Afterward, the colonies were photographed using a camera-mounted wide-field microscope and quantified by the Image-Pro Plus software (v.6.1) program (Media Cybernetics, Rockville, MD, USA).

### 4.4. Cell Apoptosis Assessment

Cells were seeded in 100 mm dishes and treated with vehicle, 5, 10, or 20 μM DFO for 72 h. Cells were subjected to staining directly without fixing. After staining with Annexin V for apoptosis or Propidium Iodide for cell cycle analysis, cells were analyzed using a BD FACSCalibur Flow Cytometer (BD Biosciences, San Jose, CA, USA).

### 4.5. Western Blot

Cell pellets were lysed using chilled NP-40 cell lysis buffer (50 mM Tris pH 8.0, 150 mM NaCl, 1% NP-40, protease inhibitor cocktail, phosphatase inhibitor cocktail, and 1 mM PMSF) and incubated over ice for 1 h. Next, the tubes were centrifuged at 14,000 rpm for 20 min, and the supernatant fractions were harvested as total cellular protein extracts. After quantifying the protein concentration using a BCA kit (Solarbio, Beijing, China), the total cellular protein extracts were resolved by SDS-PAGE. The gels were transferred to polyvinylidene fluoride (PVDF) membranes in transfer buffer and subsequently incubated with antibodies against the indicated protein markers. Blots were washed and then incubated with the appropriate HRP-linked secondary IgG. The protein bands were visualized using the enhanced chemiluminescence (ECL) detection reagent (Cytiva, Amersham Pl, Little Chalfont, Bucks, UK).

### 4.6. Ex Vivo Pull-Down Assay

Cell lysates (500 μg) were incubated with DFO-Sepharose 4 B (or Sepharose 4 B only as a control) beads (50 μL; 50% slurry) in the reaction buffer (50 mM Tris-HCl, 150 mM NaCl, 5 mM EDTA, 1 mM dithiothreitol, 0.2 mM PMSF and 0.01% NP-40, 20× protease inhibitor). After incubating overnight at 4 °C with gentle rocking, the beads were washed three times with washing buffer (50 mM Tris-HCl, 150 mM NaCl, 5 mM EDTA, 1 mM DTT, 0.01% NP-40, and 0.2 mM PMSF), and the binding was visualized by Western blot.

### 4.7. Surface Plasmon Resonance (SPR)

The binding affinity of DFO with TAOK1 protein was assessed using a Biacore T200 instrument (Cytiva, Bjorkgatan, Uppsala, Sweden). A CM5 chip (Cat#BR-1005-30) immobilized with TAOK1 (20 μg/mL) was equilibrated with PBS solution. Gradient concentrations of DFO were perfused at a 30 μL/min flow rate to the system to achieve equilibrium between binding and dissociation. The compound–protein reaction was plotted as response units versus time. The binding–dissociation equilibrium constant (KD) was calculated using the BIA evaluation software (v.3.0).

### 4.8. In Vitro Kinase Assay

The in vitro TAOK1 kinase assay was performed by mixing 50 ng of active TAOK1 kinase, 400 ng of MBP-GST proteins, and 200 μM of ATP reagent with the indicated concentrations of DFO (0, 2.5, 5, and 10 μM). The mixture was incubated at 30 °C for 30 min, and the reaction was terminated with 25 μL of 3 × SDS loading buffer. The activity of TAOK1 kinase was assessed by Western blot using a p-ser/thr antibody. GST antibody was used as a loading control which indicated the loading amount of MBP.

### 4.9. Virtual Screening

TAOK1’s 3D structure was predicted by alphafold2. Binding pockets were predicted with sitemap and the ATP one was selected for virtual screening. For TAOK1 protein preparation, Schrödinger’s Protein Preparation Wizard (2023-2) was used. Bond orders were assigned, hydrogens were added, and missing side chains were fixed using Prime. Water and cofactors were removed, and residues were optimized at pH 7.0 using PROPKA. OPLS_4 minimized energy and focused on hydrogens till RMSD hit 0.3 Å. TargetMol’s L1010FDA library (3158cpds) was used. Its 2D sdf file was imported. Ligprep made 3D substrates and set coordinates based on OPLS_4. Epik found stereoisomers and protonation states. Around the ATP pocket, the docking grid was centered on the predicted site, with the outer box sized according to the predicted volume and an inner box of 10 Å. Schrödinger’s Virtual Screening Workflow performed molecular docking using Ligprep’s 3D structures. Virtual screening had 4 modes: (1) HTVS kept all ligand stereoisomers, making one conformation per distinct state. For L1010, the top 50% highest-scoring compounds advanced. (2) SP kept well-scoring stereoisomers with one conformation per state, and the top 20% proceeded. (3) XP kept the best-scoring compounds, with one conformation per state, and the top 100 passed. (4) MM-GBSA rescored the final compounds and removed duplicates.

### 4.10. Enzyme-Linked Immunosorbent Assay

The cell supernatant and mouse serum were used for iron measurement analysis using transferrin and ferritin ELISA kits (Abcam, Cambridge, UK). The analysis of transferrin and ferritin was performed according to the manufacturer’s protocol. Briefly, 50 μL of cell supernatant or serum was added to the corresponding wells and mixed with an equal volume of antibody cocktail. After 1 h of incubation at room temperature, the plate was washed with 1× washing buffer PT. Then, 100 μL of substrate was added to each well, followed by 100 μL of stop solution. The OD value at 450 nm was detected and recorded using a microplate reader.

### 4.11. Lentiviral Infection

Lenti-293T cells were transfected with shMock or shTAOK1 plasmids as well as packaging vectors (*pMD2G* and *psPAX2*) using the Simple-Fect Transfection Reagent (Signaling Dawn Biotech, Wuhan, Hubei, China). The virus particle-enriched medium was harvested by filtration using a 0.22 μm filter at 36 h and then stored at −80 °C. The cells were infected by the virus-enriched medium supplemented with 8 μg/mL polybrene (Millipore, Billerica, MA, USA) for 24 h. Then, cells were selected with 2 μg/mL puromycin for 48 h.

### 4.12. Multi-Omics Sequencing

Multi-omics analyses (proteomics and phosphoproteomics) were performed by the Applied Protein Technology Co., Ltd., Shanghai, China (https://bio-cloud.aptbiotech.com/plus/#/home/index, accessed on 13 December 2021). Specifically, proteins were extracted using SDT buffer (4% SDS, 100 mM Tris-HCl, 1 mM DTT, pH 7.6) and quantified with a BCA kit (Bio-Rad, Hercules, CA, USA). After trypsin digestion, peptides were desalted, concentrated, and reconstituted in 40 µL of 0.1% (*v*/*v*) formic acid. The protein samples were separated on a 12.5% SDS-PAGE gel and visualized by Coomassie Brilliant Blue R-250 staining. Phosphopeptide enrichment was carried out with the High-Select™ Fe-NTA Phosphopeptide Enrichment Kit (Thermo Scientific, Waltham, MA, USA) according to the manufacturer’s instructions. LC-MS/MS analysis was conducted on a timsTOF Pro mass spectrometer (Bruker, Billerica, MA, USA) coupled to a NanoElute system (Bruker Daltonics, Billerica, MA, USA) with 60/120/240 min runs. Finally, the MS raw data from each sample were combined and analyzed using MaxQuant 1.5.3.17 for protein identification and quantification.

### 4.13. Patient-Derived Xenograft (PDX) Mouse Model

LEG170 and HEG63 PDX tumor masses were subcutaneously implanted into the backs of six- to eight-week-old severe combined immunodeficiency (SCID) mice (Cyagen Biosciences Inc., Guangzhou, China). When the average tumor volume reached approximately 100 mm^3^, mice were divided into 3 groups: A vehicle group and 20 mg/kg and 40 mg/kg DFO treatment groups (n = 8 for LEG170 and n = 5 for HEG63). DFO was administered by oral gavage once daily for 39 (LEG170) or 22 (HEG63) days. The tumor volume was calculated using the following formula: Tumor volume (mm^3^) = (length × width × height × 0.52). Mice were monitored until tumors reached approximately 1.0 cm^3^ total volume, at which time the mice were euthanized. The animal experiments were approved by the Ethics Committee of Zhengzhou University (Zhengzhou, Henan, China) (NO: ZZUIRB-2023-010).

### 4.14. Hematoxylin–Eosin (H&E) Staining and Immunohistochemical (IHC) Analysis

The obtained tumor tissues were dehydrated by gradient ethanol and transparent treatment with xylene. The paraffin blocks were embedded with the paraffin solution. Paraffin-embedded sections with thicknesses of 5 μm were prepared for subsequent experiments. For H&E staining, hematoxylin was used for visualizing the nucleus, and eosin was used for the staining of cytosol. For IHC analysis, the specimens were blocked with 5% goat serum and incubated overnight at 4 °C with antibodies to detect Ki-67, p53, and TAOK1. DAB (3,3′-diaminobenzidine) staining was used to visualize related protein markers after incubation with the corresponding HRP-linked secondary antibody. Next, sectioned tissues were dehydrated using a graded series of ethanol and xylene before mounting them under glass coverslips. The staining was photographed using a camera-mounted wide-field microscope and analyzed with the Image-Pro Plus software.

### 4.15. Statistical Analysis

Student’s t-test or one-way ANOVA was calculated using the Graph Pad Prism v7 software (San Diego, CA, USA) to conduct statistical analysis for single or multi-comparisons. The value of *p* < 0.05 was used as the criterion for statistical significance. The data are shown as mean values ± standard error (S.E.) for animal experiments, or standard deviation (S.D.) for the other experiments.

## 5. Conclusions

We demonstrated that Deferasirox (DFO) effectively inhibits the progression of ESCC by directly binding to and suppressing the kinase activity of TAOK1, independent of its iron-chelating properties. DFO induces apoptosis through the activation of the p53 signaling pathway. Moreover, DFO significantly reduces tumor growth in ESCC PDX models in vivo, supporting its potential as an effective therapeutic option (Figure 7). In summary, these findings provide a strong rationale for repurposing DFO in the treatment of ESCC and highlight TAOK1 as a promising target in kinase-driven cancers.

## Data Availability

The original contributions presented in this study are included in the article/Supplementary Materials. Further inquiries can be directed to the corresponding author(s).

## Supplementary Materials

The supporting information can be downloaded at: https://www.mdpi.com/article/10.3390/ijms26041524/s1.
