# Supplementary material for: Deferasirox Targets TAOK1 to Induce p53-Mediated Apoptosis in Esophageal Squamous Cell Carcinoma
Source: Int J Mol Sci. 2025 Feb 11;26(4):1524. doi: 10.3390/ijms26041524 (PMC11855378; doi:10.3390/ijms26041524)
Supplement: Supplementary file 1 [file ijms-26-01524-s001.zip › Supplementary figures and figure legends-revised-20250209.pdf]

A

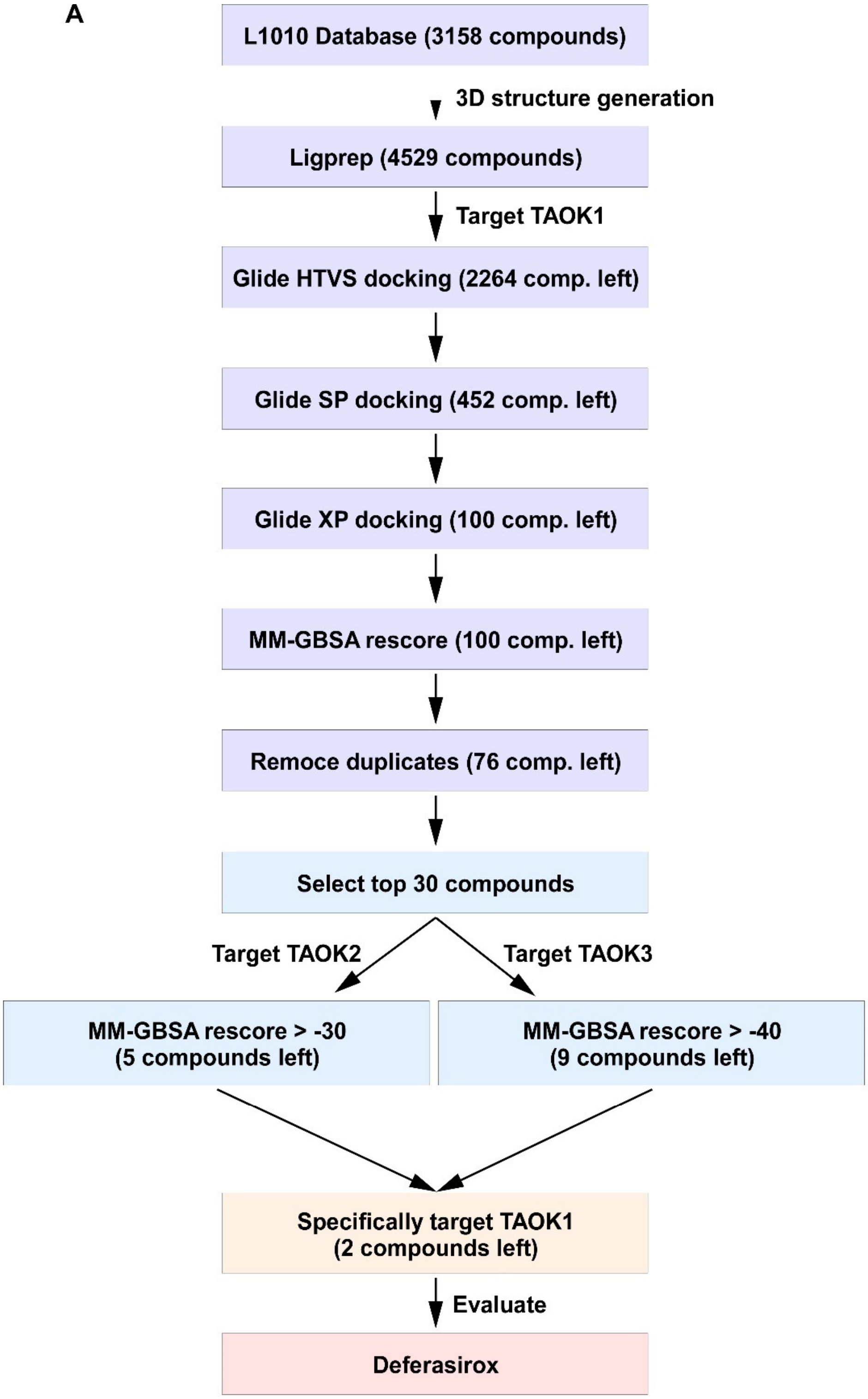

B

The docking results of Deferasirox and TAOKs kinases

Protein kinase	UniPort ID	MM-GBSA dG Bind	XP Gscore
TAOK1	Q7L7X3	-42.39 kcal/mol	-8.12
TAOK2	Q9UL54	-25.47 kcal/mol	-8.552
TAOK3	Q9H2K8	-38.59 kcal/mol	-6.894

Supplementary Figure S1: The virtual screening workflow and results of TAOKs. (A) The flowchart delineates the sequential steps involved in the screening process for small molecule compounds that specifically target the TAOK1 protein kinase. (B) Docking Results of Deferasirox and TAOKs Kinases.

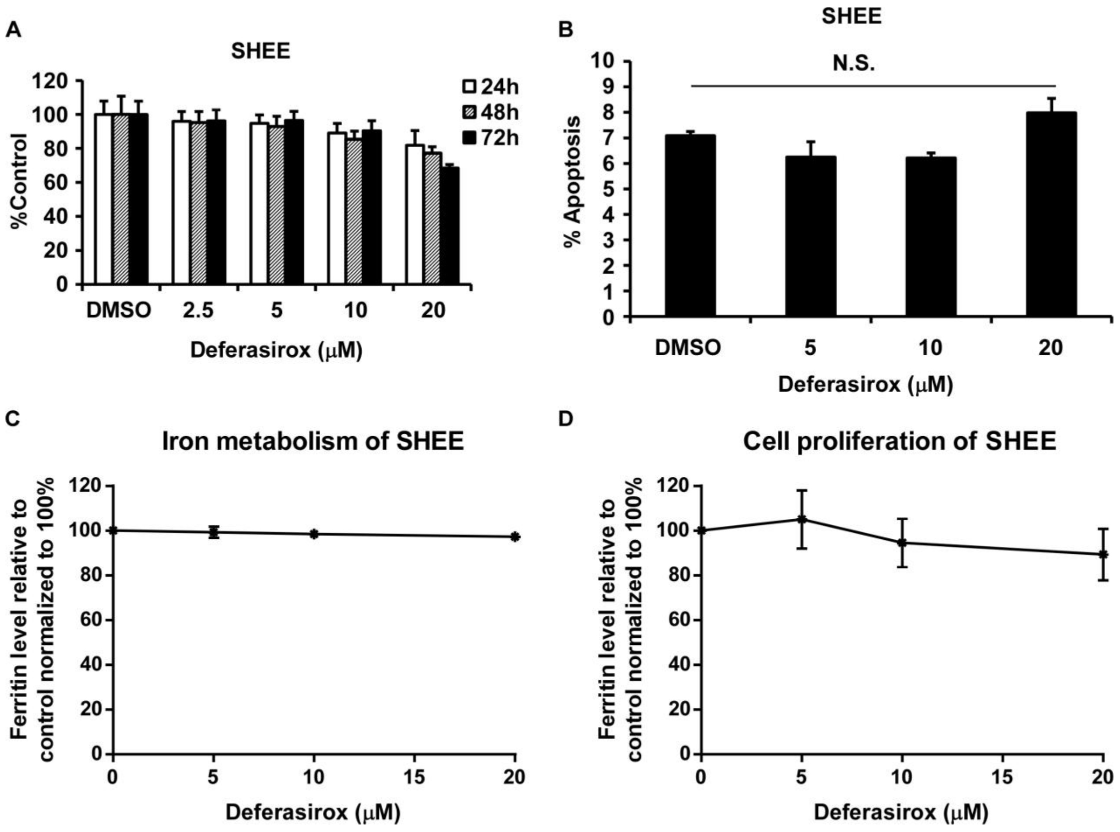

Supplementary Figure S2: Cytotoxicity effect of DFO on normal esophageal epithelial SHEE cells. (A) Effect of DFO on SHEE cell growth was detected using MTT assay at 24, 48, and 72 h. DMSO, 2.5, 5, 10 and 20 μM DFO were adopted for measurements. (B) Effect of DFO on SHEE cell apoptosis. N.S. indicates that DFO has no effect on SHEE cell apoptosis. (C) DFO (0~20μM) had no effect on iron metabolism in SHEE cells. (D) DFO (0~20μM) had no effect on cell proliferation in SHEE cells.

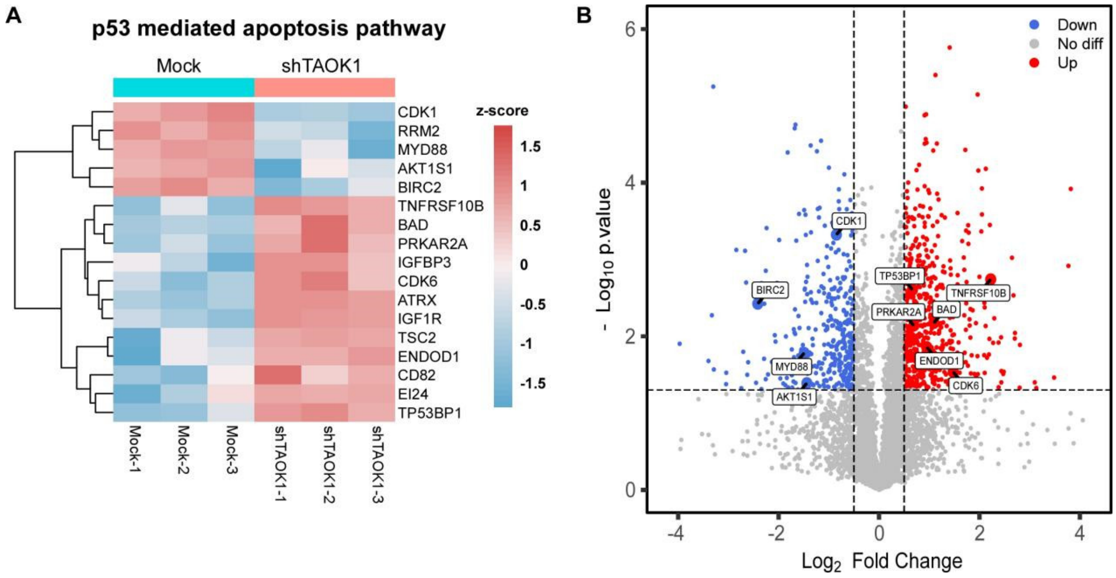

Supplementary Figure S3: The expression changes of genes in p53-related pathway in proteomic sequencing. (A) The heatmap displays the upregulated and downregulated genes in the p53-related pathway following TAOK1 knockdown. (B) The volcano plot illustrates the upregulated and downregulated genes in the proteomic sequencing data after TAOK1 knockdown.

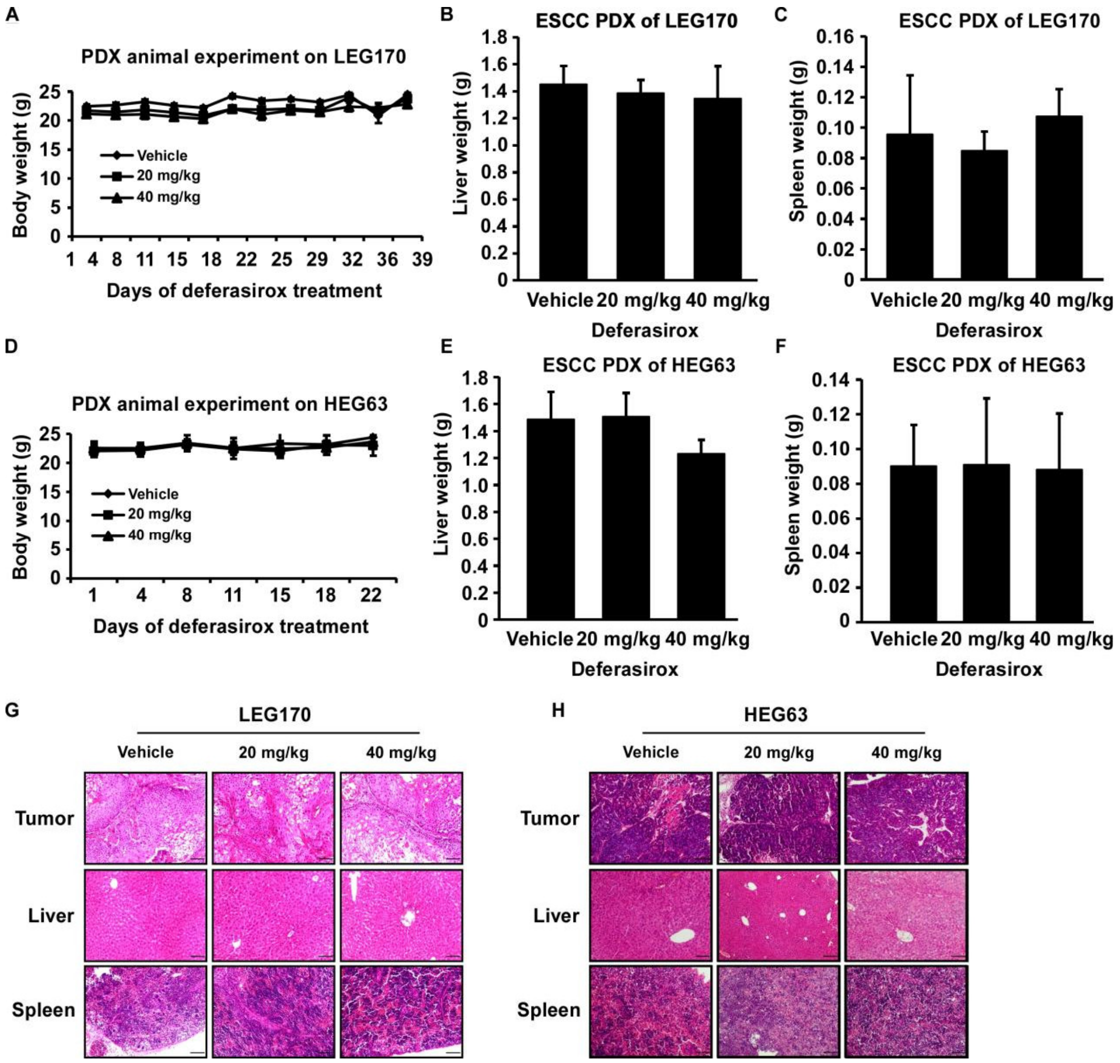

Supplementary Figure S4: DFO exhibits no slide effect in mice. (A, D) Body weights of mice (LEG170 and HEG63) treated daily with vehicle or DFO were plotted over 39 and 22 days. (B, C, E, F) The liver and spleen weights of the vehicle or DFO-treated mice (LEG170 and HEG63) were measured and summarized. (G, H) H&E staining of liver, spleen, and tumor specimens are illustrated (100× magnification). N.S. indicates no significant change in DFO-treated groups compared to controls.

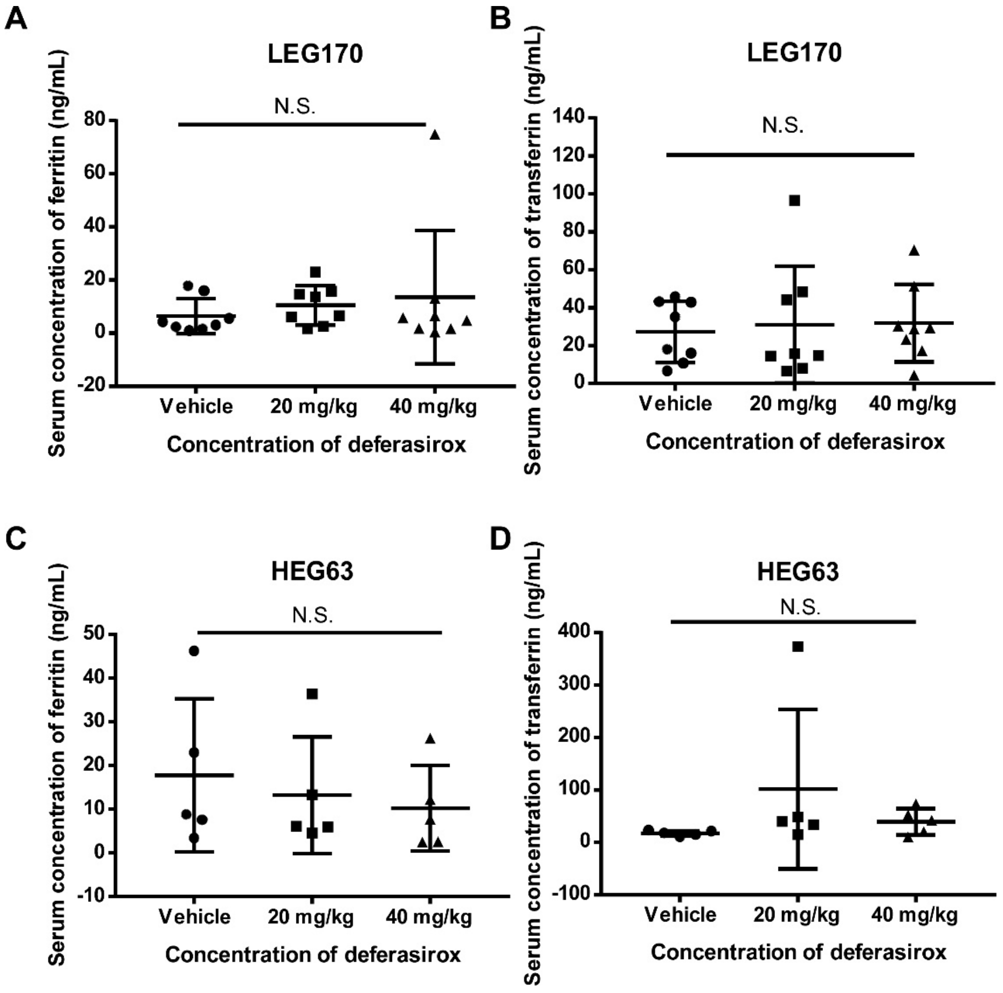

Supplementary Figure S5: DFO exhibits week inhibitory effect on ESCC iron metabolism levels. The effects of different concentrations of deferasirox on the serum concentrations of ferritin and transferrin in LEG170 and HEG63 cases. (A, C) The changes in the serum concentration of ferritin in LEG170 and HEG63 treated with different concentrations of deferasirox (Vehicle, 20 mg/kg, 40 mg/kg), respectively. (B, D) The changes in the serum concentration of transferrin after corresponding treatments. Data points represent the mean values, error bars indicate standard deviations, and N.S. denotes no significant difference.
