# Supplementary material for: Deferasirox Targets TAOK1 to Induce p53-Mediated Apoptosis in Esophageal Squamous Cell Carcinoma
Source: Int J Mol Sci. 2025 Feb 11;26(4):1524. doi: 10.3390/ijms26041524 (PMC11855378; doi:10.3390/ijms26041524)
Supplement: Supplementary file 1 [file ijms-26-01524-s001.zip › Supplementary file-docking list.pdf]

ID	Name	MM-GBSA dG Bind(TAOK1)	ID	MM-GBSA dG Bind(TAOK2)	ID	MM-GBSA dG Bind(TAOK3)
T2717	Difamilast	-60.22	T0866	-61.61	T6S1966	-60.71
T1528	Azasetron hydrochloride	-57.46	T0970	-53.5	T0970	-59.77
T0970	Racanisodamine	-54.54	T0342	-52.05	T8149	-57.81
T0173	Nylidrin hydrochloride	-52.43	T8149	-51.66	T2885	-56.53
T30027	Amosulalol	-51.08	T2485	-51.07	T12829	-53.14
T1186	Ifenprodil Tartrate	-49.65	T12829	-47.43	T2485	-52.17
TJS2190	Nordihydroguaiaretic acid	-49.51	T0154	-46.97	T2717	-49.97
T12829	Salmeterol	-48.7	T1027	-46.37	T2836	-49.1
T2885	Esculin	-48.64	T23132	-46.36	T1528	-48.79
T0430	Imidazolidinyl urea	-48.54	T2717	-43.73	T0154	-48.1
T23358	Sitagliptin phosphate	-48.08	T1528	-43.27	T3060	-47.74
T8149	Dobutamine hydrochloride	-47.98	T6301	-43.03	T1186	-46.85
T3278	Piribedil	-46.38	T0173	-42.79	T6301	-44.4
T6S1966	Curculigoside	-45.35	T1186	-42.52	T0173	-43.89
T2836	Isorhamnetin	-45.14	T6S1966	-40.44	T1646	-43.8
T1027	Luteolin	-44.84	T2885	-39.64	T30027	-43.43
T39043	Tenofovir amibufenamide	-44.62	T4285	-38.83	T0866	-43.16
T2175	Apigenin	-44.6	TJS2190	-38.11	T0430	-43.09
T2397	Topiroxostat	-44.42	T3278	-37.43	T2397	-42.2
T2778	(+)-Catechin Hydrate	-43.88	T3060	-37.39	TJS2190	-41.19
T0866	Propafenone	-43.84	T0430	-36.84	T23358	-40.28
T0154	Nebivolol hydrochloride	-43.36	T30027	-36.44	T2778	-39.83
T1646	Famciclovir	-43.34	T2778	-34.89	T4285	-39.5
T23132	Pentamidine dihydrochloride	-43.1	T2836	-32.82	T1457	-38.59
T0342	Carvedilol phosphate	-42.82	T2175	-32.17	T23132	-38.52
T3060	Fasudil hydrochloride	-42.44	T1646	-28.17	T39043	-38.26
T1457	Deferasirox	-42.39	T23358	-26.89	T0342	-36.95
T2485	Baricitinib	-41.66	T2397	-26.29	T2175	-36.12
T6301	Tosedostat	-41.32	T1457	-25.47	T1027	-33.83
T4285	Crisaborole	-40.87	T39043	-14.63	T3278	-32.61

postscript:	Virtual screening was carried out using the L1010FDA library (L1010 - FDA Approved & Pharmacopeial Drug Library - 3158 compounds) provided by TargetMol Company. After removing duplicates, 76 compounds were finally obtained. Since the TAOKs family includes TAOK1, TAOK2 and TAOK3, in order to find small molecule compounds that specifically target the TAOK1 protein kinase, we selected the top 30 small molecule compounds ranked by MM-GBSA dG Bind value and conducted molecular docking with both TAOK2 protein kinase and TAOK3 protein kinase, and then screened for small molecule inhibitors that had poor docking results and specifically targeted TAOK1. Among the small molecules docked with TAOK2 protein kinase, we screened for those with an MM-GBSA dG Bind value greater than -30 kcal/mol, and a total of five were obtained. As for the docking results of small molecules with TAOK3, since the worst MM-GBSA dG Bind value was still less than -30 kcal/mol, we relaxed the criteria and screened for small molecules with an MM-GBSA dG Bind value greater than -40 kcal/mol, and a total of nine were obtained. We took the intersection of the results of the two molecular docking operations and obtained Tenofovir amibufenamide and Deferasirox as candidate drugs that specifically target the TAOK1 protein kinase. After manual evaluation of these two drugs, since Tenofovir amibufenamide is an antiviral drug and there have been no research reports on its anti-tumor effects for the time being, while there have been relevant research reports on Deferasirox in cancer, Deferasirox was finally determined as the target drug for our research.
ID:	The numbers of small molecule compounds in the L1010 - FDA Approved & Pharmacopeial Drug Library - 3158 compoun
Name:	Drug name
MM-GBSA dG Bin	Free energy of binding between target proteins and small molecule drugs
Yellow:	Drugs selected according to the docking results of thirty kinds of small molecules with TAOK2 protein kinase
Green:	Drugs selected according to the docking results of thirty kinds of small molecules with TAOK3 protein kinase

Targeting TAOK1

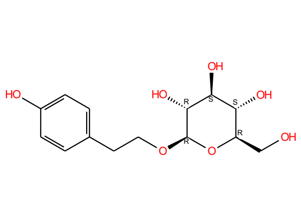

title	T2717
MMGBSA d(	-60.22
XP GScore	-10.173

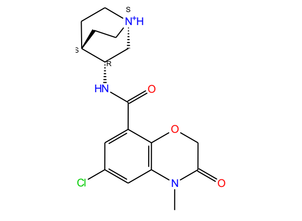

title	T1528
MMGBSA d(	-57.46
XP GScore	-8.37

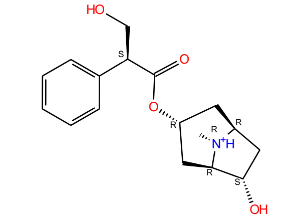

title	T0970
MMGBSA d(	-54.54
XP GScore	-7.012

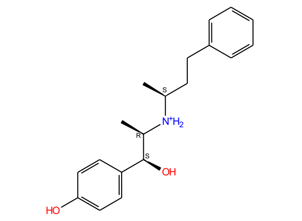

title	T0173
MMGBSA d(	-52.43
XP GScore	-6.752

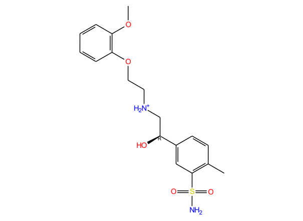

title	T30027
MMGBSA d(	-51.08
XP GScore	-8.017

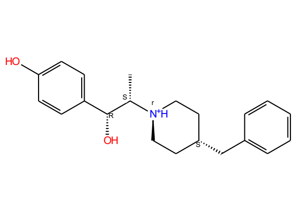

title	T1186
MMGBSA d(	-49.65
XP GScore	-6.822

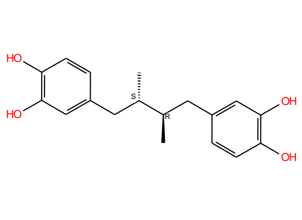

title	TJS2190
MMGBSA d(	-49.51
XP GScore	-10.405

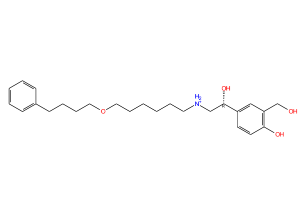

title	T12829
MMGBSA d(	-48.7
XP GScore	-8.535

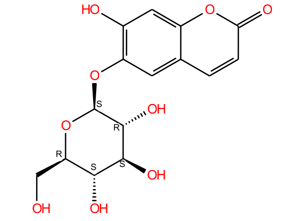

title	T2885
MMGBSA d(	-48.64
XP GScore	-9.521

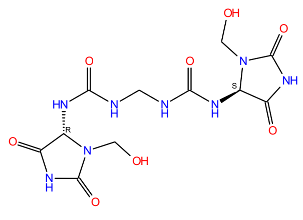

title	T0430
MMGBSA d(	-48.54
XP GScore	-6.869

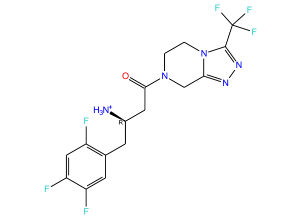

title	T23358
MMGBSA d(	-48.08
XP GScore	-6.893

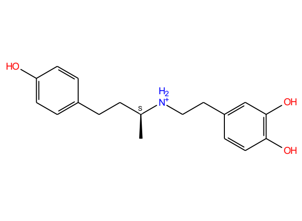

title	T8149
MMGBSA d(	-47.98
XP GScore	-8.53

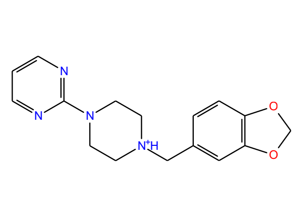

title	T3278
MMGBSA d(	-46.38
XP GScore	-6.968

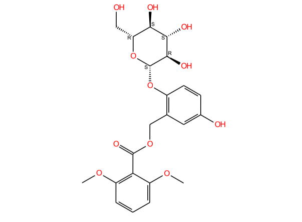

title	T6S1966
MMGBSA d(	-45.35
XP GScore	-7.352

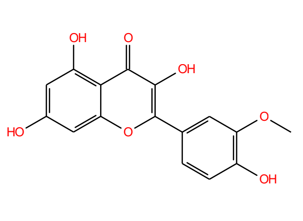

title	T2836
MMGBSA d(	-45.14
XP GScore	-8.501

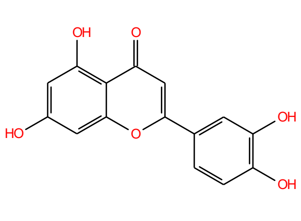

title	T1027
MMGBSA d(	-44.84
XP GScore	-9.226

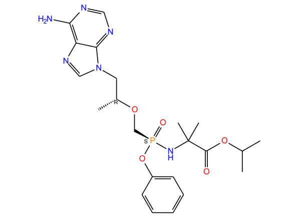

title	T39043
MMGBSA d(	-44.62
XP GScore	-9.975

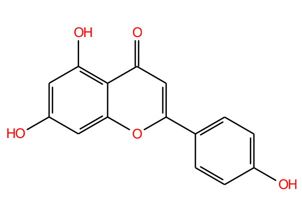

title	T2175
MMGBSA d(	-44.6
XP GScore	-8.281

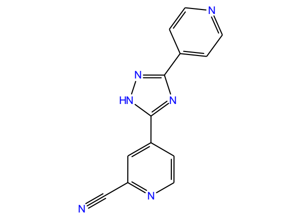

title	T2397
MMGBSA d(	-44.42
XP GScore	-6.935

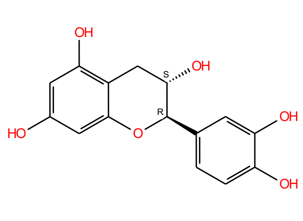

title	T2778
MMGBSA d(	-43.88
XP GScore	-7.766

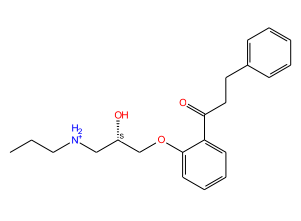

title	T0866
MMGBSA d(	-43.84
XP GScore	-7.009

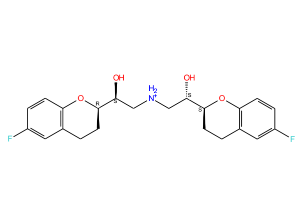

title	T0154
MMGBSA d(	-43.36
XP GScore	-7.643

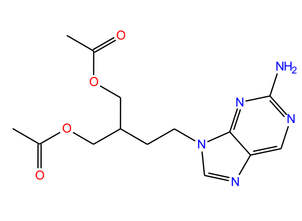

title	T1646
MMGBSA d(	-43.34
XP GScore	-7.561

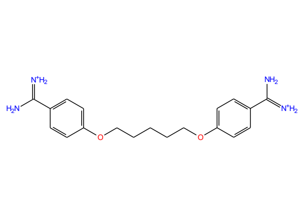

title	T23132
MMGBSA d(	-43.1
XP GScore	-7.467

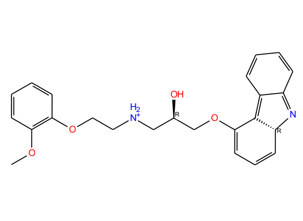

title	T0342
MMGBSA d(	-42.82
XP GScore	-7.409

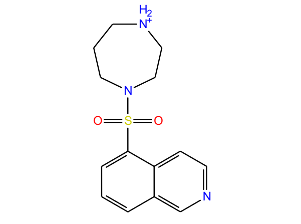

title	T3060
MMGBSA d(	-42.44
XP GScore	-8.568

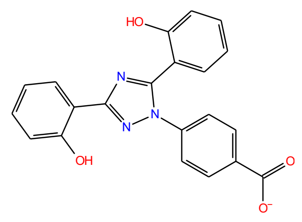

title	T1457
MMGBSA d(	-42.39
XP GScore	-8.12

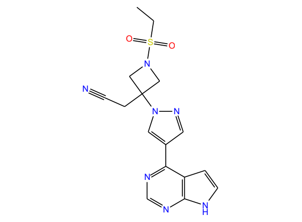

title	T2485
MMGBSA d(	-41.66
XP GScore	-8.177

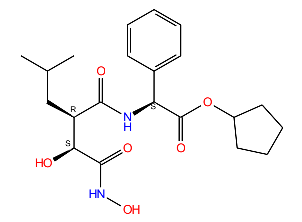

title	T6301
MMGBSA d(	-41.32
XP GScore	-7.452

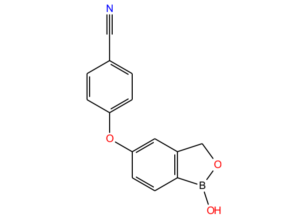

title	T4285
MMGBSA d(	-40.87
XP GScore	-6.804

Targeting TAOK1

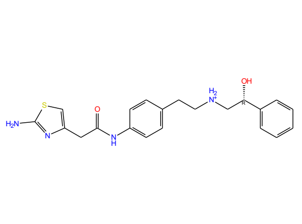

title	T1671
MMGBSA d(	-40.85
XP GScore	-9.475

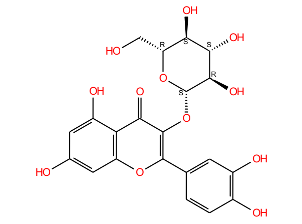

title	T5S0754
MMGBSA d(	-40.72
XP GScore	-11.295

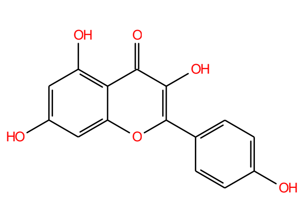

title	T2177
MMGBSA d(	-40.35
XP GScore	-7.543

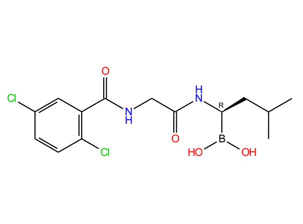

title	T2122
MMGBSA d(	-40.03
XP GScore	-8.369

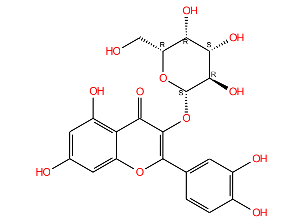

title	T2844
MMGBSA d(	-39.65
XP GScore	-12.297

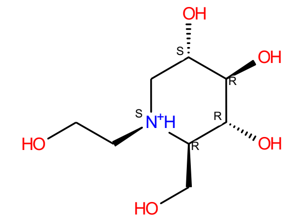

title	T1529
MMGBSA d(	-37.98
XP GScore	-6.979

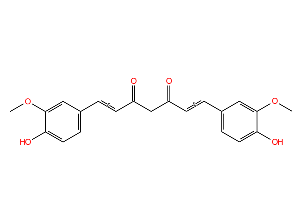

title	T1516
MMGBSA d(	-37.69
XP GScore	-8.491

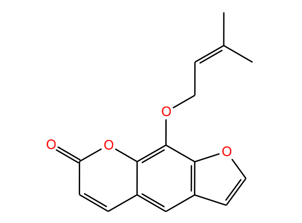

title	T2845
MMGBSA d(	-37.65
XP GScore	-7.135

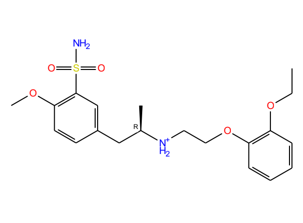

title	T0210
MMGBSA d(	-37.61
XP GScore	-7.545

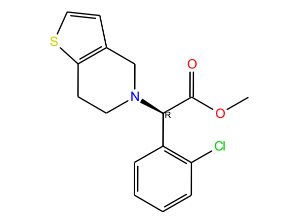

title	T0182L2
MMGBSA d(	-36.79
XP GScore	-8.055

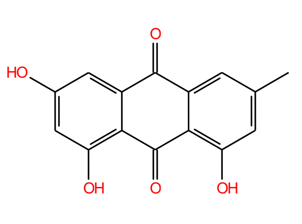

title	T2869
MMGBSA d(	-36.36
XP GScore	-7.534

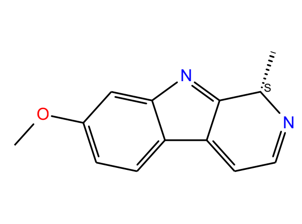

title	T2811
MMGBSA d(	-36.26
XP GScore	-7.512

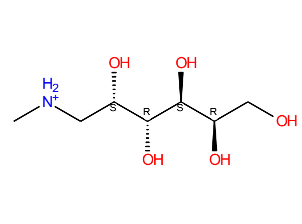

title	T7582
MMGBSA d(	-36.07
XP GScore	-8.338

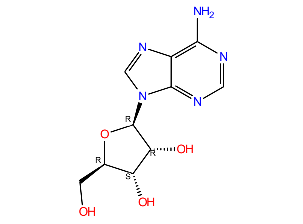

title	T0853
MMGBSA d(	-35.77
XP GScore	-6.842

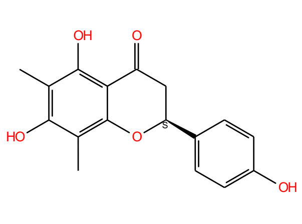

title	T6S0525
MMGBSA d(	-34.13
XP GScore	-6.976

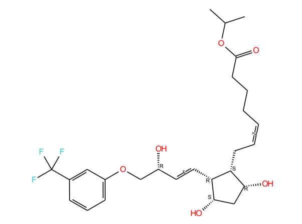

title	T5841
MMGBSA d(	-34.06
XP GScore	-6.968

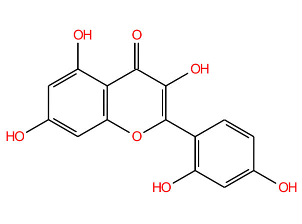

title	T2835
MMGBSA d(	-33.7
XP GScore	-8.802

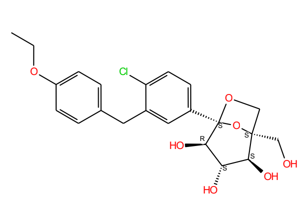

title	T4999
MMGBSA d(	-33.57
XP GScore	-10.488

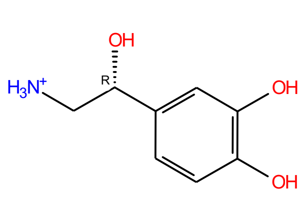

title	T1064
MMGBSA d(	-33.5
XP GScore	-8.439

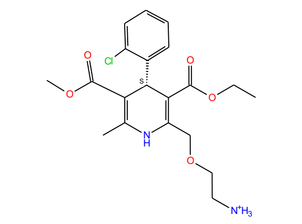

title	T4284
MMGBSA d(	-33.36
XP GScore	-7.153

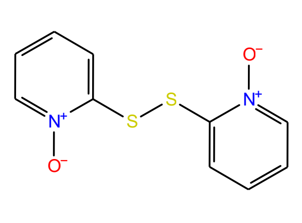

title	T7969
MMGBSA d(	-33.28
XP GScore	-7.565

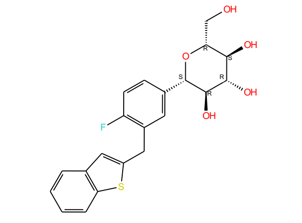

title	T2385
MMGBSA d(	-33.09
XP GScore	-8.406

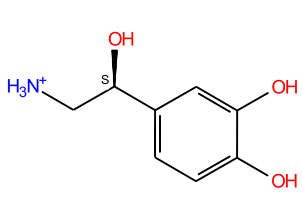

title	T21066
MMGBSA d(	-33.01
XP GScore	-8.375

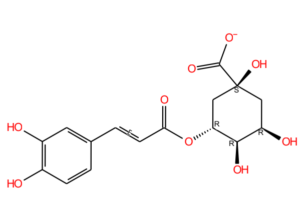

title	T2805
MMGBSA d(	-32.56
XP GScore	-9.757

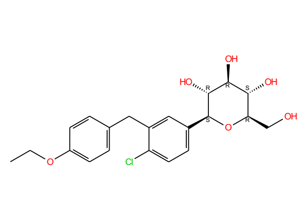

title	T4460
MMGBSA d(	-32.26
XP GScore	-9.961

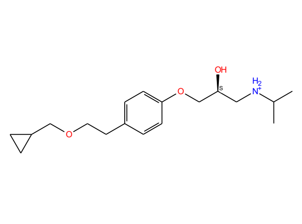

title	T6565
MMGBSA d(	-32.02
XP GScore	-7.937

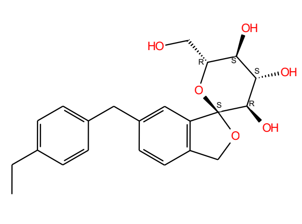

title	T5016
MMGBSA d(	-30.88
XP GScore	-8.241

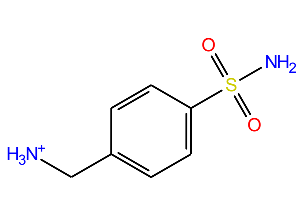

title	T8122
MMGBSA d(	-28.74
XP GScore	-6.984

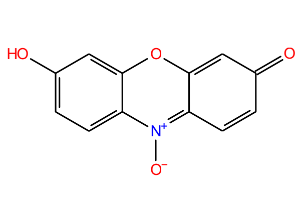

title	T7650
MMGBSA d(	-28.44
XP GScore	-7.828

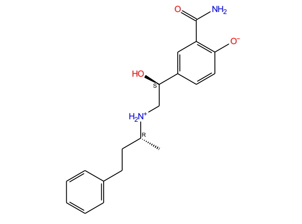

title	T0348
MMGBSA d(	-27.11
XP GScore	-7.009

Targeting TAOK1

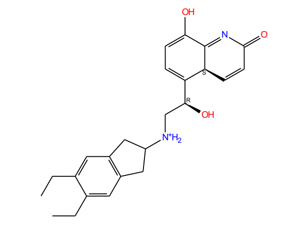

title	T2320
MMGBSA d(	-26.79
XP GScore	-7.937

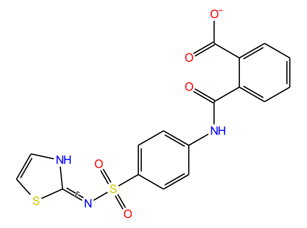

title	T0512
MMGBSA d(	-26.19
XP GScore	-7.178

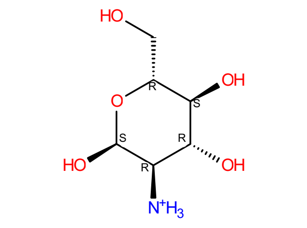

title	T2941
MMGBSA d(	-24.6
XP GScore	-9.175

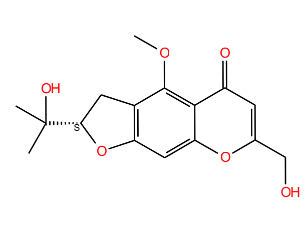

title	T3383
MMGBSA d(	-23.83
XP GScore	-6.865

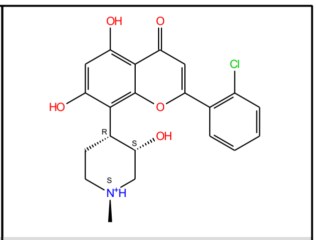

title	T6837
MMGBSA d(	-21.44
XP GScore	-6.804

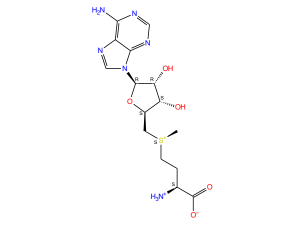

title	T6752
MMGBSA d(	-20.54
XP GScore	-7.798

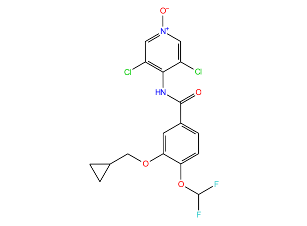

title	T4462
MMGBSA d(	-20.23
XP GScore	-8.047

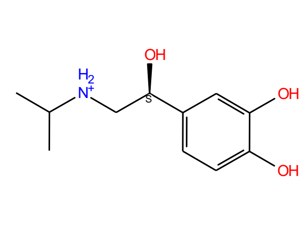

title	T1056
MMGBSA d(	-17.12
XP GScore	-6.9

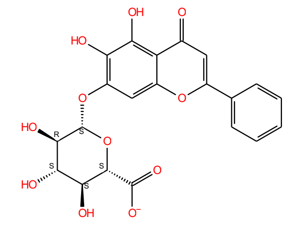

title	T2775
MMGBSA d(	-13.63
XP GScore	-9.844

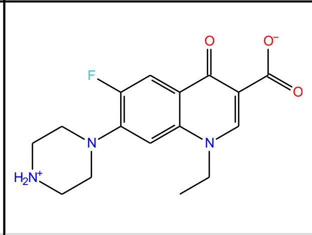

title	T1306
MMGBSA d(	-12.87
XP GScore	-7.193

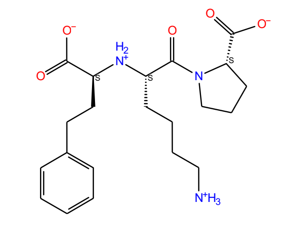

title	T0706
MMGBSA d(	-12.33
XP GScore	-8.285

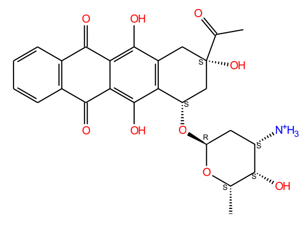

title	T6010
MMGBSA d(	-12.18
XP GScore	-7.861

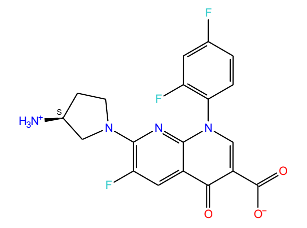

title	T9319
MMGBSA d(	-6.99
XP GScore	-7.024

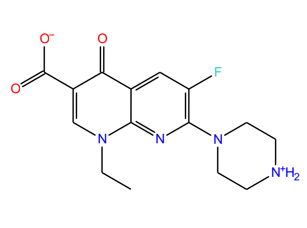

title	T0717
MMGBSA d(	-5.15
XP GScore	-6.907

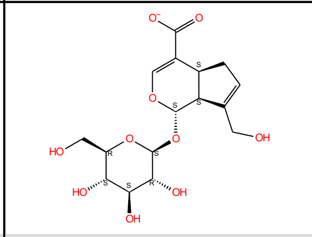

title	T2136
MMGBSA d(	-4.85
XP GScore	-8.194

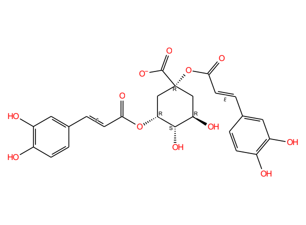

title	T6S1529
MMGBSA d(	-1.37
XP GScore	-8.506

Targeting TAOK2

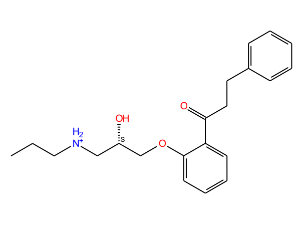

title	T0866
MMGBSA d(	-61.61
XP GScore	-8.406

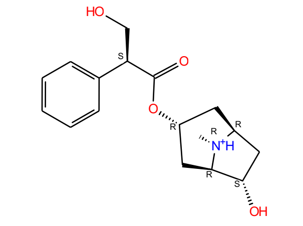

title	T0970
MMGBSA d(	-53.5
XP GScore	-7.972

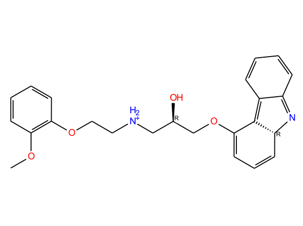

title	T0342
MMGBSA d(	-52.05
XP GScore	-9.238

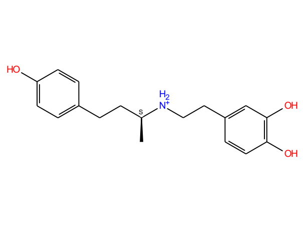

title	T8149
MMGBSA d(	-51.66
XP GScore	-8.563

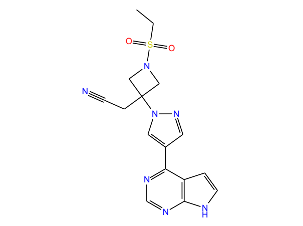

title	T2485
MMGBSA d(	-51.07
XP GScore	-8.624

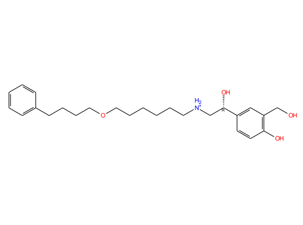

title	T12829
MMGBSA d(	-47.43
XP GScore	-7.323

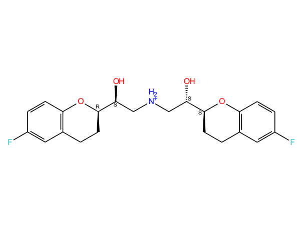

title	T0154
MMGBSA d(	-46.97
XP GScore	-8.356

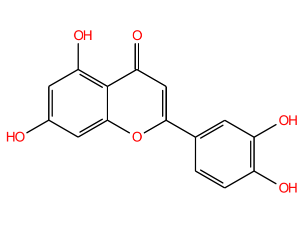

title	T1027
MMGBSA d(	-46.37
XP GScore	-9.517

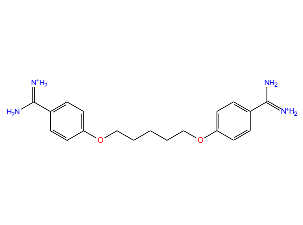

title	T23132
MMGBSA d(	-46.36
XP GScore	-7.754

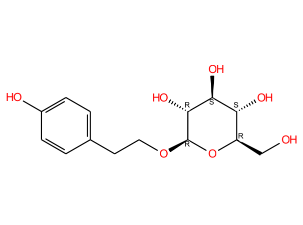

title	T2717
MMGBSA d(	-43.73
XP GScore	-9.82

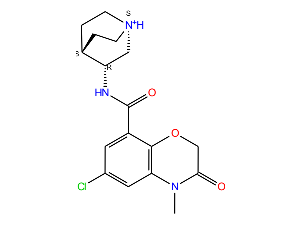

title	T1528
MMGBSA d(	-43.27
XP GScore	-6.977

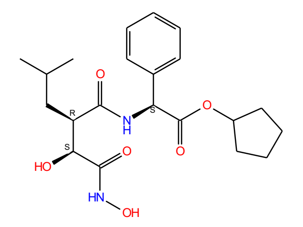

title	T6301
MMGBSA d(	-43.03
XP GScore	-7.584

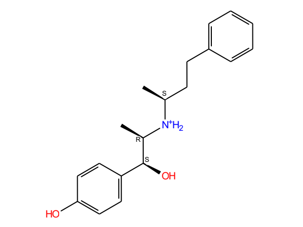

title	T0173
MMGBSA d(	-42.79
XP GScore	-8.145

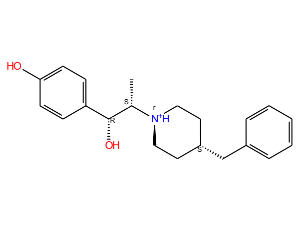

title	T1186
MMGBSA d(	-42.52
XP GScore	-4.065

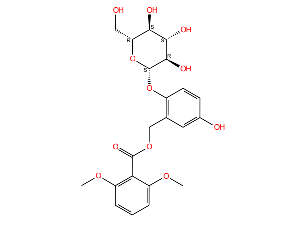

title	T6S1966
MMGBSA d(	-40.44
XP GScore	-11.384

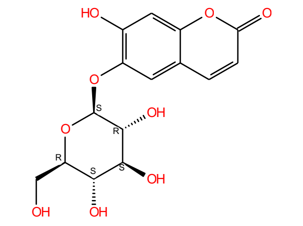

title	T2885
MMGBSA d(	-39.64
XP GScore	-11.904

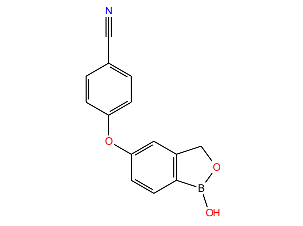

title	T4285
MMGBSA d(	-38.83
XP GScore	-6.788

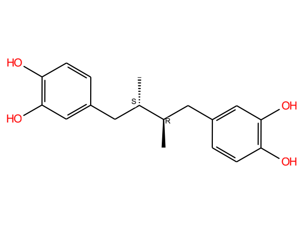

title	TJS2190
MMGBSA d(	-38.11
XP GScore	-10.284

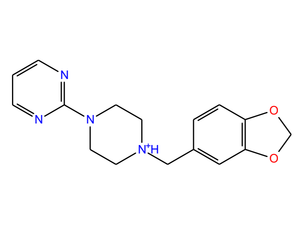

title	T3278
MMGBSA d(	-37.43
XP GScore	-5.73

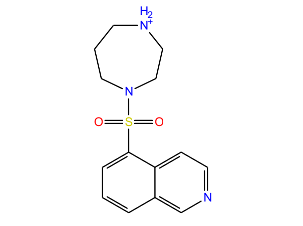

title	T3060
MMGBSA d(	-37.39
XP GScore	-5.42

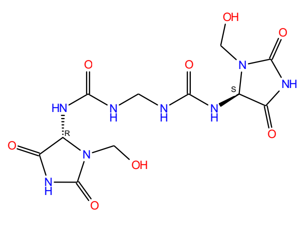

title	T0430
MMGBSA d(	-36.84
XP GScore	-7.385

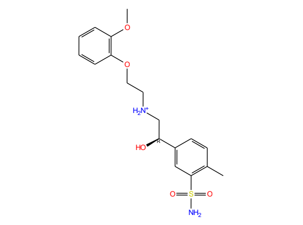

title	T30027
MMGBSA d(	-36.44
XP GScore	-6.614

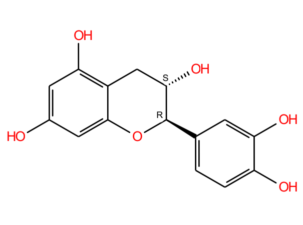

title	T2778
MMGBSA d(	-34.89
XP GScore	-8.698

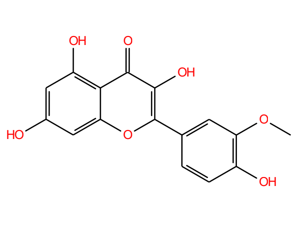

title	T2836
MMGBSA d(	-32.82
XP GScore	-10.119

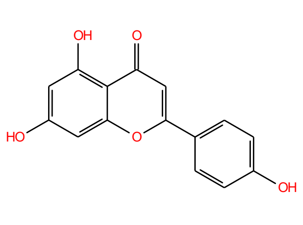

title	T2175
MMGBSA d(	-32.17
XP GScore	-8.534

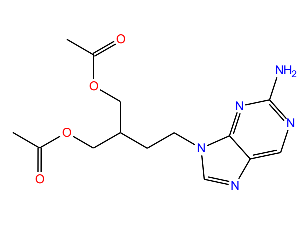

title	T1646
MMGBSA d(	-28.17
XP GScore	-5.618

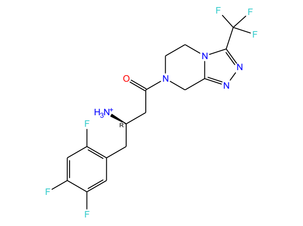

title	T23358
MMGBSA d(	-26.89
XP GScore	-9.006

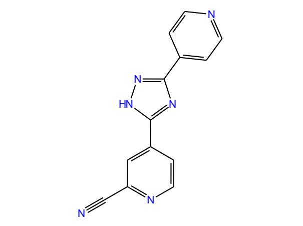

title	T2397
MMGBSA d(	-26.29
XP GScore	-3.19

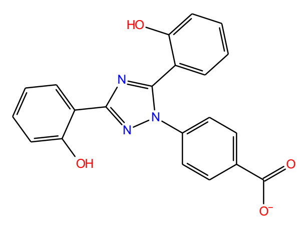

title	T1457
MMGBSA d(	-25.47
XP GScore	-8.552

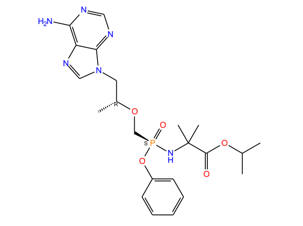

title	T39043
MMGBSA d(	-14.63
XP GScore	-8.46

Targeting TAOK3

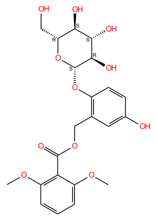

title	T6S1966
MMGBSA d(	-60.71
XP GScore	-10.977

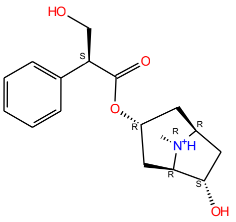

title	T0970
MMGBSA d(	-59.77
XP GScore	-1.599

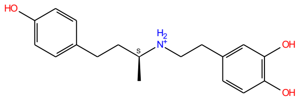

title	T8149
MMGBSA d(	-57.81
XP GScore	-7.833

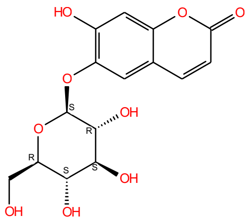

title	T2885
MMGBSA d(	-56.53
XP GScore	-10.878

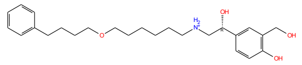

title	T12829
MMGBSA d(	-53.14
XP GScore	-7.462

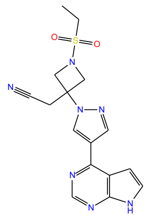

title	T2485
MMGBSA d(	-52.17
XP GScore	-9.195

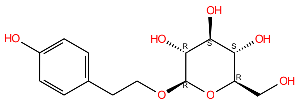

title	T2717
MMGBSA d(	-49.97
XP GScore	-10.397

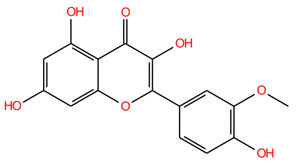

title	T2836
MMGBSA d(	-49.1
XP GScore	-9.197

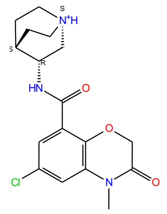

title	T1528
MMGBSA d(	-48.79
XP GScore	-8.057

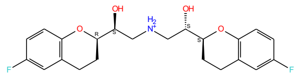

title	T0154
MMGBSA d(	-48.1
XP GScore	-8.933

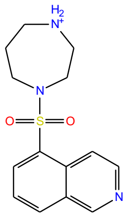

title	T3060
MMGBSA d(	-47.74
XP GScore	-8.334

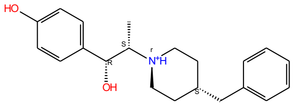

title	T1186
MMGBSA d(	-46.85
XP GScore	-6.807

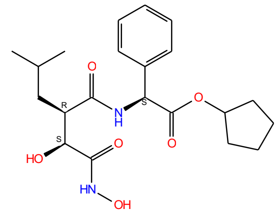

title	T6301
MMGBSA d(	-44.4
XP GScore	-7.062

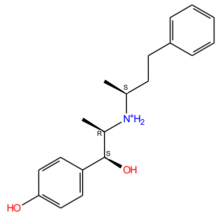

title	T0173
MMGBSA d(	-43.89
XP GScore	-7.188

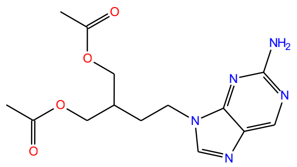

title	T1646
MMGBSA d(	-43.8
XP GScore	-9.366

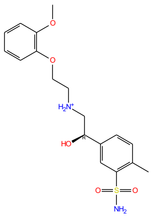

title	T30027
MMGBSA d(	-43.43
XP GScore	-5.387

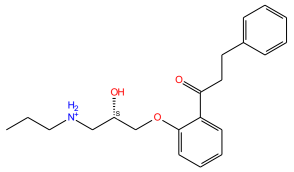

title	T0866
MMGBSA d(	-43.16
XP GScore	-7.966

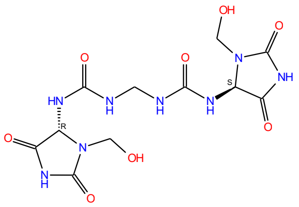

title	T0430
MMGBSA d(	-43.09
XP GScore	-9.507

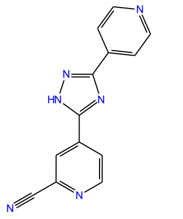

title	T2397
MMGBSA d(	-42.2
XP GScore	-8.139

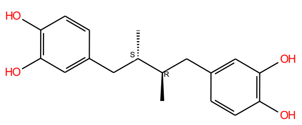

title	TJS2190
MMGBSA d(	-41.19
XP GScore	-9.861

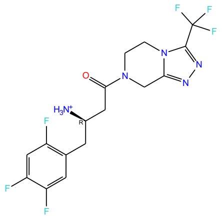

title	T23358
MMGBSA d(	-40.28
XP GScore	-6.545

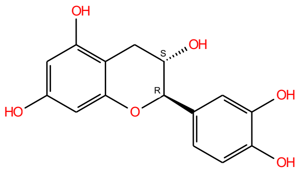

title	T2778
MMGBSA d(	-39.83
XP GScore	-9.454

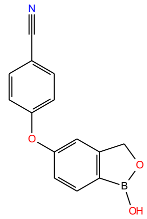

title	T4285
MMGBSA d(	-39.5
XP GScore	-8.057

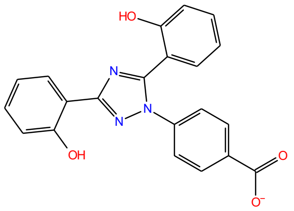

title	T1457
MMGBSA d(	-38.59
XP GScore	-6.894

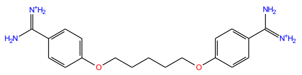

title	T23132
MMGBSA d(	-38.52
XP GScore	-5.657

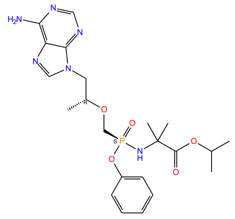

title	T39043
MMGBSA d(	-38.26
XP GScore	-10.575

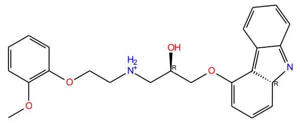

title	T0342
MMGBSA d(	-36.95
XP GScore	-9.846

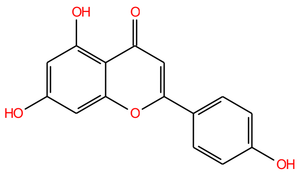

title	T2175
MMGBSA d(	-36.12
XP GScore	-10.589

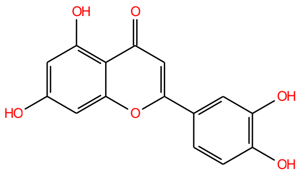

title	T1027
MMGBSA d(	-33.83
XP GScore	-11.499

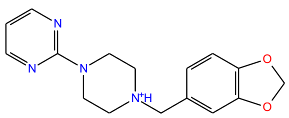

title	T3278
MMGBSA d(	-32.61
XP GScore	-4.66
